# Behavioural and functional evidence revealing the role of RBFOX1 variation in multiple psychiatric disorders and traits

**DOI:** 10.1038/s41380-022-01722-4

**Published:** 2022-08-10

**Authors:** Aet O’Leary, Noèlia Fernàndez-Castillo, Gabriela Gan, Yunbo Yang, Anna Y. Yotova, Thorsten M. Kranz, Lena Grünewald, Florian Freudenberg, Ester Antón-Galindo, Judit Cabana-Domínguez, Anais Harneit, Janina I. Schweiger, Kristina Schwarz, Ren Ma, Junfang Chen, Emanuel Schwarz, Marcella Rietschel, Heike Tost, Andreas Meyer-Lindenberg, Christiane A. Pané-Farré, Tilo Kircher, Alfons O. Hamm, Demian Burguera, Nina Roth Mota, Barbara Franke, Susann Schweiger, Jennifer Winter, Andreas Heinz, Susanne Erk, Nina Romanczuk-Seiferth, Henrik Walter, Andreas Ströhle, Lydia Fehm, Thomas Fydrich, Ulrike Lueken, Heike Weber, Thomas Lang, Alexander L. Gerlach, Markus M. Nöthen, Georg W. Alpers, Volker Arolt, Stephanie Witt, Jan Richter, Benjamin Straube, Bru Cormand, David A. Slattery, Andreas Reif

**Affiliations:** 1 Department of Psychiatry, Psychosomatic Medicine and Psychotherapy, University Hospital, Goethe University Frankfurt, Frankfurt am Main, Germany; 2 Department of Neuropscyhopharmacology, Institute of Chemistry, University of Tartu, Tartu, Estonia; 3 Department de Genètica, Microbiologia i Estadística, Facultat de Biologia, Universitat de Barcelona, and Institut de Biomedicina de la Universitat de Barcelona (IBUB); Barcelona, Catalonia, Spain; Centro de Investigación Biomédica en Red de Enfermedades Raras (CIBERER), Instituto de Salud Carlos III (ISCIII); Madrid, Spain; Institut de Recerca Sant Joan de Déu (IR-SJD), Esplugues de Llobregat, Barcelona, Catalonia Spain; 4 Department of Psychiatry and Psychotherapy, Central Institute of Mental Health, Medical Faculty Mannheim, University of Heidelberg, Mannheim, Germany; 5 Department of Psychiatry and Psychotherapy and Center for Mind, Brain and Behavior - CMBB, University of Marburg, Marburg, Germany; 6 Institute of Cell Biology and Neuroscience, Faculty of Biosciences, Goethe University Frankfurt, Frankfurt am Main, Germany; 7 Department of Biological and Clinical Psychology/Psychotherapy, University of Greifswald, Greifswald, Germany; 8 Department of Zoology, Charles University, Prague, Czech Republic; 9 Department of Human Genetics and Department of Psychiatry, Donders Institute for Brain, Cognition and Behaviour, Radboud University Medical Center, Nijmegen, Netherlands; 10 Mainz University Medical Center, Institute of Human Genetics, Mainz, Germany; 11 Department of Psychiatry and Psychotherapy, Charité - University Medicine Berlin, Berlin, Germany; 12 Department of Psychology, Humboldt-Universität zu Berlin, Berlin, Germany; 13 Department of Psychiatry, Psychosomatic Medicine and Psychotherapy, University of Würzburg, Würzburg, Germany; 14 Christoph-Dornier-Foundation for Clinical Psychology, Institute for Clinical Psychology Bremen; Bremen, Germany and Department for Psychology & Methods, Jacobs University Bremen, Bremen, Germany; 15 Clinical Psychology and Psychotherapy, University of Cologne, Cologne, Germany; 16 Institute of Human Genetics, School of Medicine and University Hospital Bonn, University of Bonn, Bonn, Germany; 17 Department of Psychology, School of Social Sciences, University of Mannheim, Mannheim, Germany; 18 Department of Psychiatry and Psychotherapy, University of Münster, Münster, Germany; 19 Department of Genetic Epidemiology in Psychiatry, Central Institute of Mental Health, Medical Faculty Mannheim, University of Heidelberg, Mannheim, Germany; 20 Department of Experimental Psychopathology, University of Hildesheim, Hildesheim, Germany

**Keywords:** Autism spectrum disorders, Genetics, Schizophrenia

## Abstract

Common variation in the gene encoding the neuron-specific RNA splicing factor RNA Binding Fox-1 Homolog 1 (*RBFOX1*) has been identified as a risk factor for several psychiatric conditions, and rare genetic variants have been found causal for autism spectrum disorder (ASD). Here, we explored the genetic landscape of RBFOX1 more deeply, integrating evidence from existing and new human studies as well as studies in *Rbfox1* knockout mice. Mining existing data from large-scale studies of human common genetic variants, we confirmed gene-based and genome-wide association of *RBFOX1* with risk tolerance, major depressive disorder and schizophrenia. Data on six mental disorders revealed copy number losses and gains to be more frequent in ASD cases than in controls. Consistently, *RBFOX1* expression appeared decreased in post-mortem frontal and temporal cortices of individuals with ASD and prefrontal cortex of individuals with schizophrenia. Brain-functional MRI studies demonstrated that carriers of a common *RBFOX1* variant, rs6500744, displayed increased neural reactivity to emotional stimuli, reduced prefrontal processing during cognitive control, and enhanced fear expression after fear conditioning, going along with increased avoidance behaviour. Investigating *Rbfox1* neuron-specific knockout mice allowed us to further specify the role of this gene in behaviour. The model was characterised by pronounced hyperactivity, stereotyped behaviour, impairments in fear acquisition and extinction, reduced social interest, and lack of aggression; it provides excellent construct and face validity as an animal model of ASD. In conclusion, convergent translational evidence shows that common variants in *RBFOX1* are associated with a broad spectrum of psychiatric traits and disorders, while rare genetic variation seems to expose to early-onset neurodevelopmental psychiatric disorders with and without developmental delay like ASD, in particular. Studying the pleiotropic nature of RBFOX1 can profoundly enhance our understanding of mental disorder vulnerability.

## Introduction

Mental disorders are characterised by substantial heritability but have a complex genetic architecture contributed by common genetic variants of individually small effects and rare variants of intermediate or large effects [1]. A high degree of comorbidity and substantial genetic correlation among psychiatric diseases point to pleiotropic effects of etiologic factors. Genetic pleiotropy is also seen in loss-of-function variants causing rare, severe genetic syndromes, while regulatory variations are associated with milder, more frequent forms of a disorder. In the most recent Psychiatric Genomics Consortium (PGC) cross-disorder genome-wide association studies (GWAS) meta-analysis [2, 3], RNA Binding Fox-1 Homolog 1 (*RBFOX1*) was the second most pleiotropic locus and found to be associated with seven out of eight disorders analysed. Since then, GWAS on mood and anxiety disorders and neuroticism have extended the spectrum of disorders and behavioural traits involving common variants of *RBFOX1* [4–7]. Beyond these studies of common genetic variation, rare genetic variants in *RBFOX1* such as copy number variants (CNVs) and loss-of-function mutations have been related to early-onset neurodevelopmental disorders, especially autism spectrum disorder (ASD) and attention-deficit/hyperactivity disorder (ADHD) [8–13]; furthermore, case (series) reports have also implicated rare variants in *RBFOX1* in developmental delay, intellectual disability, epilepsy, and aggression [14]. *RBFOX1* encodes a splicing factor that regulates alternative splicing and the expression of large networks of genes involved in brain development [15, 16]. These data collectively implicate *RBFOX1* as one of the most relevant risk genes for psychopathology; however, neither the specific behavioural domains nor the involved neural circuits have yet been identified.

To delineate the genetic contribution of *RBFOX1* to mental disorders, we comprehensively data-mined and synthesised large-scale datasets on common and rare genetic variations in psychiatric disorders and traits. We next studied the influence of genetic variation in *RBFOX1* on human neural circuits to uncover its functional consequences. To do so, we focused on a common genetic variant, which emerged as the most promising SNP (rs6500744; risk allele: C) in a previous study [14]. Given the association of common variants in *RBFOX1* with mood and anxiety disorders and neuroticism (see above), we investigated the effect of rs6500744 on circuits underlying emotion processing, fear conditioning, and executive functioning using functional magnetic resonance imaging (fMRI), and a test assessing standardised fear reactions and avoidance behaviour towards an aversive stimulus, the Behavioural Avoidance Task (BAT [17]). The functional consequences of the SNPs and CNVs in *RBFOX1* are yet unknown. However, given the decreased expression of *RBFOX1* observed in post-mortem studies of ASD and SCZ patients, and the observed over-abundance of *RBFOX1* CNV-deletions in (early-onset) mental disorders, we reasoned that loss of RBFOX1 function might underlie at least part of the observed associations. We, therefore, generated a neuron-specific *Rbfox1* heterozygous (HET) and homozygous (KO) knockout mouse line to determine the behavioural consequences of decreased *Rbfox1* expression in the brain.

## Materials and methods

### Common and rare genetic risk variants in *RBFOX1* in psychiatric phenotypes

The contribution of common variants (MAF > 0.01) in the *RBFOX1* gene to psychiatric disorders or related behavioural traits was assessed through SNP-based and gene-based association studies using GWAS summary statistics from previous studies (Supplementary Table 1). In total, eleven psychiatric conditions or traits were investigated: attention deficit-hyperactivity disorder (ADHD), aggression (AGG), anorexia (ANO), anxiety (ANX), autism spectrum disorder (ASD), bipolar disorder (BIP), major depressive disorder (MDD), obsessive-compulsive disorder (OCD), risk tolerance (RT), schizophrenia (SCZ) and Tourette’s syndrome (TS), and the cross-disorder meta-analysis of eight of them (CD-MA). Further information about the SNP- and gene-based analyses can be found in Supplementary Methods.

CNVs in *RBFOX1* were collected from publicly available data from the above disorders or traits (in patients and in controls when reported), either in published papers (until April 2020) or databases (DECIPHER, https://decipher.sanger.ac.uk; ClinVar, https://www.ncbi.nlm.nih.gov/clinvar; ISCA, http://dbsearch.clinicalgenome.org/search/). To inspect the overlap between CNVs identified in patients and putative cis-regulatory elements we used epigenetic data from ENCODE (https://www.encodeproject.org/) from seven neural tissues and brain-related Hi-C data from 3DIV to identify interactions with the first distal promoter. We performed burden analysis for *RBFOX1* CNVs in 18 out of 34 studies where information in controls was available using PLINK v.1.07 considering CN loss and CN gains separately as well as both together.

### Expression of *RBFOX1* in brain samples of ASD and SCZ patients

Alterations in the expression of *RBFOX1* in the brain were assessed using transcriptomic data from post-mortem brain samples of ASD and SCZ patients, compared to controls, using publicly available human datasets, either in GEO (http://www.ncbi.nlm.nih.gov/geo) or published articles (Supplementary Table 7). *RBFOX1* expression was explored in different brain areas, including the hippocampus, cerebellum, or cortex, depending on the dataset.

### *RBFOX1* rs6500744 in functional MRI

#### Genotyping

DNA was extracted from whole blood according to standard procedures for all participants. Then, genome-wide SNP genotyping was performed using a standard GWAS chip (PsychChip, Illumina Human610-Quad BeadChip [Illumina, Inc., San Diego]). Based on this genome-wide chip, genotype information for the rs6500744 *RBFOX1* SNP was retrieved for each individual using plink (http://zzz.bwh.harvard.edu/plink/). The observed genotype distribution of rs6500744 did not deviate from the Hardy-Weinberg equilibrium (Flanker/Go-NoGo: *p* = 0.826 [C/C carrier: *n* = 71, C/T carrier: *n* = 158, T/T carrier: *n* = 95]; Face matching: *p* = 0.821 [C/C carrier: *n* = 70, C/T carrier: *n* = 154, T/T carrier: *n* = 89]; computed based on the CRAN R-package, https://cran.r-project.org/web/packages/Hardy-Weinberg /index.html;). Analogous to previous imaging genetic studies on common genetic risk variants for psychiatric disorders [18–21], we compared brain activation for risk-allele carrier (C/C and C/T) to no-risk allele carrier (T/T carrier) of rs6500744 (as proposed by [14]).

#### Flanker/Go-NoGo and face matching tasks

##### Sample

We included the data of 324 (Flanker/Go-NoGo task) and 313 (Face matching task) healthy adults of European ancestry who have been recruited as healthy controls within the framework of a multi-site imaging genetics study assessing the intermediate phenotypes of psychiatric disorders such as depression, schizophrenia, and bipolar disorder (for previous work, see refs. [19, 22–26]). Data collection was carried out at the Central Institute of Mental Health in Mannheim, at the Medical Faculty of the University of Bonn, and the Charité University Medicine in Berlin. All participants provided a whole-blood sample for DNA extraction and underwent a well-established implicit emotion processing paradigm (face matching task [27]) and a Flanker/Go-NoGo task [28] during fMRI. All participants provided written informed consent for study protocols approved by the Ethics committees of the Medical Faculty of Mannheim at the Ruprecht-Karls-University in Heidelberg, the Medical Faculty of the University of Bonn, and the Charité University Medicine in Berlin. General exclusion criteria for the healthy controls were a lifetime history of significant general medical, psychiatric, or neurological disorders, a family history of psychiatric disorders, current or past psychotropic pharmacological treatment, drug or alcohol use as well as head trauma (compare also, refs. [19, 26]). Detailed information about the task procedures for the Face-matching and Flanker/Go-NoGo task can be found in the Supplementary Methods.

##### fMRI

Functional MRI data were acquired on three comparable 3T TrioTim MRI scanners (Siemens, Erlangen, Germany) in Mannheim, Bonn, and Berlin using a gradient-recalled echo-planar imaging sequence (GRE-EPI) with the following MR parameters: 28 axial slices per volume, 4 mm slice thickness, 1 mm gap, TR = 2000 ms, TE = 30 ms, field of view (FOV) = 192 mm, flip angle =  80°, acquired in descending order. We acquired 135 volumes for the face matching task and 306 volumes for the Flanker/Go-Nogo task. Additionally, high-resolution T1 structural data were acquired using a 3D magnetization-prepared rapid gradient-echo (MP-RAGE) sequence with the following sequence parameters: 176 sagittal slices, 1 mm slice thickness, TR = 1570 ms, TE = 2.75 ms, TI = 800 ms, FOV = 256 mm, flip angle = 15°. Preprocessing and estimation of functional task-dependent brain activation at the subject level were carried out using the MATLAB-based statistical parametric mapping software (version SPM8, Wellcome Trust Centre for Neuroimaging, London, UK, http://www.fil.ion.ucl.ac.uk/spm/). Functional images were preprocessed for each participant. fMRI data were slice time corrected, realigned to the first image of the time series, spatially normalised to the Montreal Neurological Institute (MNI) template, resampled to 3 mm isotropic voxels, and smoothed with a 9 mm full-width at half-maximum (FWHM) Gaussian filter. Second-level analyses testing for genotype effects across participants were carried out using SPM12.

Individual brain activation maps were subjected to separate 3 (rs6500744 genotype: C/C, C/T, T/T carrier) x 2 (sex: men, women) full-factorial models including age and imaging site as regressors of no interest using SPM12 to test for the effects of rs6500744 on brain responses during response inhibition (“nogo > neutral” contrast), conflict monitoring (“incongruent > congruent” contrast), overall executive functioning (combined contrast: [nogo & incongruent] > [neutral & congruent]), and implicit emotion processing (“faces > forms”) for second level fMRI analyses. Sex was included as a between-subject factor into the full-factorial model to identify potential genotype x sex interactions in imaging space due to significant main effects of sex and sex by genotype interactions on behavioral performance (see supplementary Table 8) for both tasks and previously reported sex by genotype effects for comparable intermediate phenotypes for genetic variation of the *MAOA* gene [28]. We additionally included behavioural performance corresponding to the respective fMRI contrast (“faces > forms”; [incongruent & nogo] > [congruent & neutral]) as a regressor of no interest into second-level analyses to control for genotype effects on behavioral performance (i.e., face matching: accuracy faces—accuracy forms, Flanker Go-NoGo: accuracy [incongruent & nogo]—accuracy [congruent & neutral]). Given that altered ACC functioning during executive functioning measured with the Flanker/Go-NoGo task, during implicit emotion processing measured with the face matching task, and during fear associative learning measured with fear conditioning has previously been associated with different psychiatric risk genotypes (e.g., *MAOA*, *5-HTTLPR*, *BDNF Val*^*66*^*MET*) [18, 19, 28], we tested genotype effects in an a *priori* defined standard anatomical mask of the ACC derived from the Automated Anatomical Labeling (AAL) atlas [29]. The significance level was set to *P* < 0.05 family-wise error (FWE) corrected for multiple comparisons across all voxels within the ACC AAL mask. For fear conditioning and extinction tasks, we additionally conducted ROI analyses (*P* < 0.001 at voxel level) in the amygdala (also derived from the AAL atlas) due to its high relevance. Outside this pre-hypothesised ROI, findings were considered significant if they passed a significance threshold of *P* < 0.05 FWE corrected for multiple comparisons across the whole brain.

#### Fear conditioning and extinction

##### Sample

We included the data of 47 patients who have participated in the fMRI and genetic subprojects of the MAC multicenter psychotherapy study, which recruited in total 369 patients of European descent meeting DSM-IV (Diagnostic and Statistical Manual of Mental Disorders, Fourth Edition) criteria for PD/AG, as assessed by the Composite International Diagnostic Interview. The CONSORTs and overview of the number of participants for every substudy (genetic data, psychophysiological assessment, and fMRI) are published in ref. [30]. The study with all of its subprojects was approved by the respective local ethical committees and informed consent was obtained. Among the 47 patients with valid fMRI data sets and sequenced *RBFOX1* SNP rs6500744, the distribution of C/C, C/T, and T/T genotypes is 15, 21, and 11, respectively. Despite the restricted sample size of our clinical sample (*N* = 47), subgroup comparisons with groups of 10 to 20 subjects are expected to be robust with such a specific functional MRI task (e.g., ref. [31]). Especially group average reproducibility has been shown to be high, starting from a sample size of *N* = 10 (see Supplementary Fig. 17 in Marek et al. [32]). Detailed information on the task procedure of the fear conditioning task can be found in the Supplementary Methods.

### Behavioural avoidance task

The BAT (behavioural avoidance task) assessment was part of two study waves of the German national research network PANIC-NET. Genotypes for rs6500744 were available for a total of 333 participating patients (*n*(C/C) = 119; *n*(C/T) = 156; *n*(T/T) = 58) with a primary DSM-IV-TR diagnosis of panic disorder with agoraphobia with at least moderate disorder severity (238 females; mean age: *m* = 35.50 years, SD = 10.72; no significant differences between genotype groups). Criteria of inclusion and exclusion and patient recruitment procedure are described in detail elsewhere [33]. Diagnosis of PD/AG was established by a standardised computer-administered face-to-face Computer Assisted Personal Interview-World Health Organization-Composite International Diagnostic Interview (CAPI-WHO-CIDI) by trained and certified interviewers. All patients were free from psychotropic medication. Patients gave written informed consent after receiving a detailed description of the study. The study was approved by the Ethics Committee of the Medical Faculty of the Technical University of Dresden, which was valid for all participating centres. The highly standardised BAT procedure is described in detail elsewhere [17] and in the Supplementary Methods.

### Animals

Male *Rbfox1*^fl/fl^ (*Rbfox1*^tm1.1Dblk/J^; JAX strain 014089) and *Synapsin1*-Cre (B6.Cg-Tg(Syn1-cre)671Jxm/J; JAX strain 003966) mice were maintained on a C57Bl/6J background and housed in groups of 2–5 in standard individually ventilated cages on a 12 h light/dark cycle (lights on at 7:00) under controlled ambient conditions (21 ± 1 °C, 55 ± 5% humidity). Food and water were available *ad libitum* unless specified otherwise. To generate mice with neuron-specific deletion of *Rbfox1* (*Rbfox1*-KO), *Rbfox1*^fl/fl^ mice were crossed to mice carrying Cre-recombinase under the direction of the rat *Synapsin* I promoter (*Synapsin1*-Cre). The resulting heterozygous *Rbfox1*^fl/+^/*Synapsin1*-Cre^+/−^ (HET) female mice were crossed to *Rbfox1*^fl/fl^ (CTRL) males to produce homozygous *Rbfox1*^fl/fl^/*Synapsin1*-Cre^+/−^ (KO) offspring. *Rbfox1*^fl/fl^/ and *Rbfox1*^fl/+^/Synapsin-Cre^−/−^ mice were used as controls. While we cannot fully rule out the possibility that such breeding scheme results in *Rbfox1* deletion in non-neuronal cells (e.g., in the glial cells), the genetic make-up of this transgenic mouse line and the stability of Cre-expression being limited to neurons [34] makes this very unlikely. Male C57Bl/6J mice were used as social stimuli in the social interaction test and intruders in the aggression testing paradigm. All breeding and experimental procedures were conducted in accordance with the Directive of the European Communities Council of 24 November 1986 (86/609/EEC) and German animal welfare laws (TierSchG and TSchV) and were approved by the Darmstadt regional council (approval ID: FK/1126).

### Quantitative PCR (qPCR)

Tissue punches (1 mm, *n* = 12–18 samples per group) from different brain regions (Nucleus accumbens, striatum, ACC, septum, PVN, hippocampus, amygdala, thalamus) were isolated from coronal sections (250 µm thickness) made at −22 °C using a cryostat (Leica CM 3050 S) of brains from three *Rbfox1*-KO and four CTRL mice and stored at −80 °C. RNA was isolated using the RNeasy Plus Micro Kit (Qiagen) according to the manufacturer’s instructions. Further information on the qPCR procedure can be found in the Supplementary Methods. Expression data were calculated relative to the expression of *Sdha*, which was selected as the most stable of the four reference genes by analysis with Normfinder or *Syn1*, to normalize against a neuronal marker. Data were calculated relative to the average of the CTRL group and converted to log2.

### Mouse behavioural experiments

The experiments were conducted in three cohorts, and the behavioural batteries for each cohort are described in Supplementary Fig. 6. For habituation purposes, mice were transported to the behavioural testing room at least 45 min before testing. Experiments were performed between 9:00–14:00, with animals tested in randomised order by an experimenter blinded to the genotype (however, full blinding was not possible due to the robust phenotype of the KO mice). Behavioural apparatuses were cleaned before testing and between animals using Aerodesin 2000 (Lysoform Dr Hans Rosemann GmbH, Berlin, Germany). Detailed information on the mouse behavioural tests, including the open field (OF) test and novel object investigation, light-dark box (LDB) test, touchscreen pairwise visual discrimination task, spontaneous alternation task, pre-pulse inhibition (PPI) of the acoustic startle reflex, cued fear conditioning and extinction test, resident-intruder test, social interaction test, and marble burying test, can be found in the Supplementary Methods. Mouse behavioural data were analysed using GraphPad Prism 8.0 (GraphPad Software, San Diego, USA) and Jamovi (Version 2.2.5.0, Sydney, Australia). Unless described otherwise, data were analysed using t-tests, one- or two-way ANOVA, with repeated measures where appropriate, followed by Bonferroni *post hoc* tests. In case ANOVA assumptions of variance homogeneity and/or normality were violated, non-parametric tests (Welch’s t-test, Mann-Whitney U-test, Friedman’s test) were used. All values are presented as mean ± S.E.M.

## Results

### Genome-wide and gene-level associations between *RBFOX1* and psychiatric disorders

**Fig. 1:**
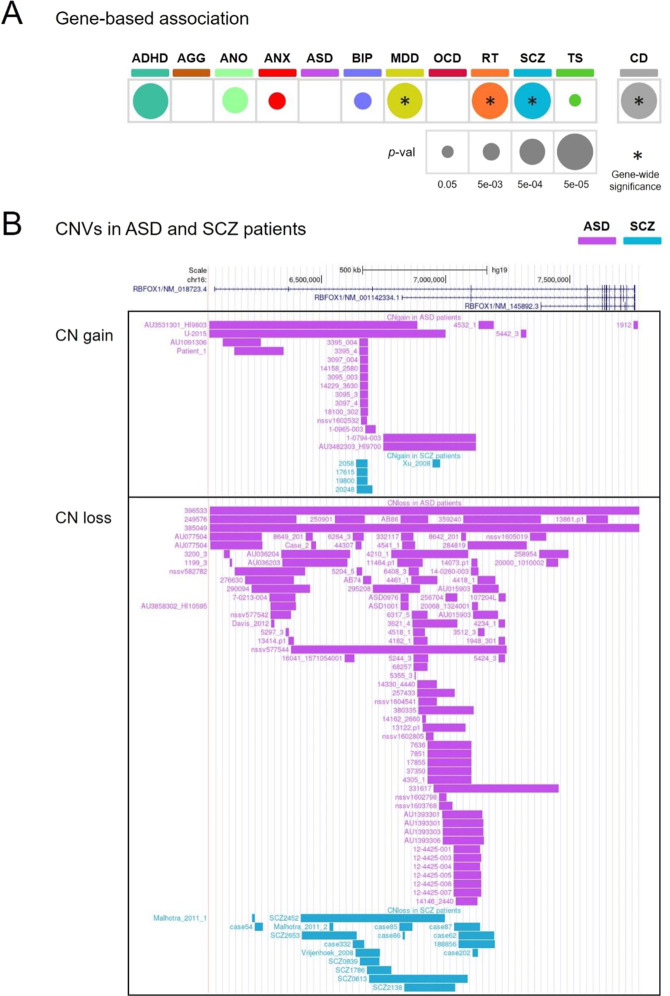
Genetic risk variants in *RBFOX1* in different psychiatric conditions and traits. **A** Common single-nucleotide variants in *RBFOX1* showed a gene-based association with most disorders and traits tested; **B** Copy number variants (CNVs) identified in ASD and SCZ patients. Top panel, copy number gains identified in ASD and SCZ patients. Bottom panel, CN losses identified in ASD and SCZ patients. Each bar represents a CNV. ADHD attention-deficit/hyperactivity disorder, AGG aggression, ANO anorexia, ANX anxiety, ASD autism spectrum disorder, BIP bipolar disorder, MDD major depressive disorder, OCD obsessive-compulsive disorder, RT risk tolerance behaviour, SCZ schizophrenia, TS Tourette’s syndrome, CD cross-disorder meta-analysis. *p*-val *p*-value.

Genome-wide associations between single nucleotide polymorphisms (SNPs) in *RBFOX1* and major depressive disorder (MDD; 38 SNPs), risk tolerance (RT; 4 SNPs), and the cross-disorder meta-analysis (CD-MA; 42 SNPs) were found in these studies (Supplementary Table 1 for sample description, data in Supplementary Table 2). At the gene level, *RBFOX1* was found to be associated with several psychiatric conditions, obtaining again gene-wide significance for MDD (*p* = 8.62e-17), RT (p = 5.6e-12), and CD-MA (*p* = 1.2e-10), but also for schizophrenia (SCZ; *p* = 7.2e-08) (Fig. 1A). Interestingly, genes associated with these disorders were significantly enriched for *RBFOX1* targets (MDD, *p* = 0.016; SCZ, *p* = 0.042; RT, *p* = 0.010; CD-MA, *p* = 0.019) (Supplementary Table 3), as it was previously shown for aggression (*p* = 3.4e-05) [35]. In line with these findings, significant associations in *RBFOX1* with other psychiatric traits or disorders, such as neuroticism, depressive symptoms, alcohol dependence, fed-up feelings or well-being spectrum, were also found using PheWAS (https://atlas.ctglab.nl/PheWAS; search terms: “RBFOX1”, domain: “psychiatric”, results sorted by *P*-value). The above evidence, therefore, highlights *RBFOX1* as a robust, replicated cross-disorder risk gene with pleiotropic effects.

### Copy number variations spanning *RBFOX1* in patients with psychiatric conditions

Next, we browsed CNVs spanning *RBFOX1* reported in patients with psychiatric conditions, identifying CNVs for six disorders/traits (in total 124 losses and 34 gains). The vast majority of CNVs were found in patients with ASD (112 CNVs), and in patients with SCZ (24 CNVs) (Fig. 1B, Supplementary Fig. 1 and Supplementary Tables 4 and 5), probably due to the larger number of CNV studies for these disorders. Across all six disorders, CNVs appeared to be 2.3 times more frequent in cases than in controls, with significant enrichment in ASD (ratio = 5:1). A significantly increased burden of CNVs was observed across studies of ADHD and ASD (Supplementary Table 5). Most of these CNVs probably affect RBFOX1 function in distinct ways; while half of them span particular exons or even the whole gene, affecting the coding sequence, many CNVs in introns overlap regions with transcription factors binding activity (such as gains in intron 2 and losses in intron 3) containing putative regulatory elements (Fig. 1B, Supplementary Figs. 1, 2, and Supplementary Tables 4, 6) and potentially altering *RBFOX1* expression. In line with this strong evidence that genetically driven variation of *RBFOX1* expression is associated with mental disorders, we synthesised knowledge of brain expression of *RBFOX1* from existing studies on *post-mortem* brain samples. Reanalysis of data from those studies showed significantly decreased *RBFOX1* mRNA levels in frontal and temporal cortices of ASD patients and prefrontal cortices of SCZ patients (Supplementary Table 7). These brain regions converge with those where the expression of *RBFOX1* is highest (Supplementary Figs. 3, 4). Taken together, common genetic variation in *RBFOX1* is robustly associated with a variety of mental disorders and behavioural traits, while rare genetic variation and reduced brain expression appear most strongly linked to neurodevelopmental disorders with onset in childhood and adolescence.

### The effects of *RBFOX1* rs6500744 on neural activation during emotion processing and executive functioning

**Fig. 2:**
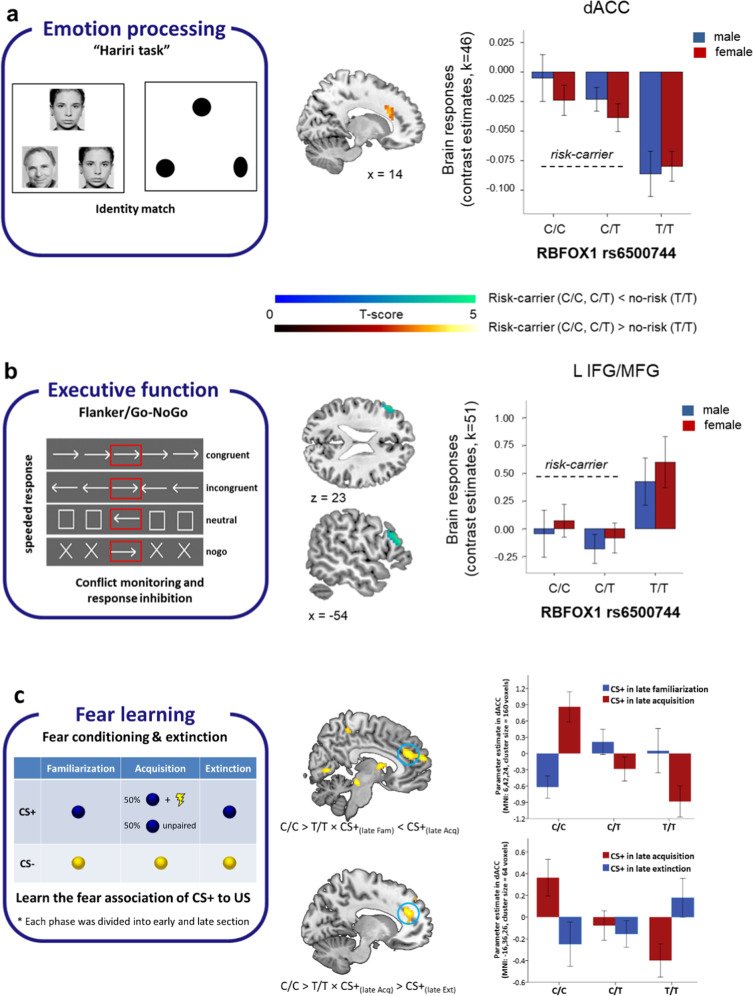
Effects of the *rs6500744 RBFOX1* genotype on brain responses during implicit emotion processing and executive functioning in healthy adults, and on fear learning in patients with panic disorder and agoraphobia. **A** left panel: Schematic overview of the face matching task. Participants had to select either one of the two faces or forms shown at the bottom of the screen that was identical to the target stimulus shown at the top of the screen. **A** right panel: C-allele carrier (C/C and C/T) showed increased brain responses in the dorsal anterior cingulate cortex (dACC) compared to those with the T/T genotype during matching faces vs. forms (faces > forms; MNI coordinate: *x* = 15, *y* = 23, z = 27, peak-voxel family-wise error-corrected [FWE] *P* = 0.010, *T* = 3.9 within bilateral ACC). **B** left panel: Schematic overview of the Flanker/Go-NoGo task. Participants had to respond to the direction of the arrow shown in the centre (red box for illustration purposes only) **B** right panel: C-allele carriers (C/C and C/T) showed reduced brain responses in the left dorsolateral prefrontal cortex (L dlPFC) compared to those homozygous for the T allele during executive functioning (contrast: [incongruent & nogo] > [congruent & neutral]; MNI coordinates: *x* = −54, *y* = 32, *z* = 21, peak-voxel *pFWE*-corrected=0.039, *T* = 4.55, across the whole-brain). Brain maps were thresholded at *p* < 0.001 uncorrected for display purposes. Error bars indicate ± 1 standard error. **C** left panel: Schematic overview of the fear conditioning and extinction task. During the acquisition phase, 50% of CS+ was paired pseudo-randomly with the US and 50% were not. Only those trials in which no US was delivered were analysed during acquisition to avoid overlap with neuron activation directly related to the presentation of the US. **C** right panel: Using ROI analysis within the ACC, homozygote risk allele carriers (C/C) compared to T/T homozygotes revealed increased activation in the dACC for CS+ after fear acquisition (CS+ in the late acquisition> CS+ in the late familiarization; cluster size = 61; peak-voxel family-wise error-corrected [FWE] *P* = 0.014, *T* = 3.87), and activation reduction for CS+ after fear extinction (CS+ in the late acquisition >CS+ in the late extinction; cluster size = 11; peak-voxel family-wise error-corrected [FWE] *P* = 0.018, *T* = 3.86). Brain maps were thresholded at *p* < 0.001 uncorrected for display purposes. Error bars indicate ± 1 standard error.

Given the role of the anterior cingulate cortex (ACC) in integrating cognition with emotion [36–38], its link with mental disorders [39], and the high level of *RBFOX1* expression in this brain area (Supplementary Fig. 3), we assessed the effects of rs6500744 on dorsal ACC (dACC) activation during implicit emotion processing and executive functioning. Region-of-interest (ROI)-analyses in 313 healthy volunteers showed an increased response of the dACC for matching fearful as well as angry faces (compared to matching geometric forms) for C-allele carriers compared to T/T carriers (Fig. 2A), suggesting increased reactivity to emotional stimuli in the target brain area. At a stringent whole-brain significance threshold, no other brain area showed a significant genotype effect during implicit emotion processing (peak-voxel family-wise error-corrected *p* < 0.05). In 324 healthy controls, ROI analyses did not reveal any significant effect of rs6500744 on dACC activation during executive functioning as measured with the Flanker/Go-NoGo task. However, whole-brain analyses revealed that C-allele carriers compared to T/T carriers showed a reduced left dorsolateral prefrontal cortex (dLPFC) response during cognitive control (contrast [incongruent & nogo] < [congruent & neutral]) (Fig. 2B). This reduced dLPFC activation during executive functioning suggests less efficient prefrontal processing during cognitive and impulse control [40, 41] that might contribute to increased impulsivity. Altered brain activation during implicit emotion processing and executive functioning as influenced by the effects of *RBFOX1* genotype may therefore underlie the increased risk for mental disorders characterised by increased emotional reactivity (e.g., MDD), impaired impulse control (e.g., ADHD, ASD, risk tolerance), and aggression, all of which are associated with *RBFOX1*.

### The effects of *RBFOX1* rs6500744 on neural activation during fear conditioning

In an independent dataset, we tested whether rs6500744 influences the neural activation in the dACC and amygdala during fear conditioning (Fig. 2C) in a sample of 47 panic disorder and agoraphobia (PD/AG) patients, again using fMRI. Compared to T/T carriers, C/C carriers revealed a significant activation enhancement in the dACC (Fig. 2C and Supplementary Table 9) for simple fear learning and a significant reduction of activation in the dACC for CS+ after fear extinction (Fig. 2C and Supplementary Table 9). ROI analyses with a threshold of *p* < 0.001 within the amygdala did not find any significant genotype differences. Since the dACC is crucial for fear appraisal [42] and expression [43–45], our findings suggest that rs6500744 C/C genotype carriers display enhanced fear expression after fear conditioning and more fear reduction after extinction training compared to T-allele carriers, which fail to demonstrate fear conditioning- and extinction-related changes in neural processing. Notably, the patients with the C/C genotype also had significantly increased depression (BDI-II) and anxiety (ASI) scores compared to T-allele carriers (Supplementary Tables 10 and 11).

### The effects of *RBFOX1* rs6500744 on fear behaviour in panic disorder/agoraphobia patients

To further investigate the effect of rs6500744 on fear behaviour, we examined its effect on avoidance during the BAT, where a behavioural and autonomous response to a fear-inducing situation is measured, in 333 PD/AG patients (Table 1). The rs6500744 C-allele was significantly and dose-dependently associated with a high frequency of avoidance behaviour (linear trend: *p* = 0.022; Table 1). This result was concurrent with observed differences between genotypes according to everyday life avoidance behaviour, assessed by clinical expert ratings (Clinical Global Index): again, avoidance increased linearly with the number of C-alleles (linear trend: *p* = 0.04, Table 1). In the 106 BAT non-avoiding patients who reported at least moderate fear during the task, the heart rate during both the anticipation and exposure phase was significantly increased relative to the recovery phase with an increasing number of C-alleles (linear trend BAT phase × genotype: *p* = 0.031, Supplementary Table 12) indicating increased autonomic threat processing. Importantly, T/T allele homozygotes did not show any heart rate modulation during the BAT. Together with the fMRI data, this suggests that rs6500744 C-allele carriers show more avoidance behaviour due to better fear learning and improved stimulus discrimination.

**Table 1:** Behavioural avoidance task: Sociodemographic and psychological characteristics of panic disorder patients with C/C, C/T and T/T *RBFOX1* rs6500744 SNP genotypes and frequency of avoidance behaviour during the task.

	C/C (*n* = 119)	C/T (*n* = 156)	T/T (*n* = 58)	*F/Chi*^*2*^
Age in years	36.00 ± 10.44	35.46 ± 10.93	34.57 ± 10.80	0.35
Female gender	87 (73%)	107 (69%)	44 (76%)	1.34
Years of education				2.04
≤8	18	19	8	
9─11	67	87	28	
≥12	34	50	22	
CGI total	5.04 ± 0.74	5.15 ± 0.80	5.03 ± 0.90	0.75
CGI avoidance	4.65 ± 1.03	4.59 ± 1.10	4.31 ± 1.29	2.13
SIGH-A	23.34 ± 7.44	22.54 ± 7.68	22.19 ± 8.19	0.56
MI alone	2.91 ± 0.84	2.92 ± 0.85	2.72 ± 0.86	1.13
PAS	27.19 ± 9.72	26.76 ± 9.59	25.17 ± 10.80	0.84
ASI	32.82 ± 11.19	30.03 ± 11.63	30.14 ± 10.34	2.29
BDI-II	16.71 ± 9.17	15.55 ± 7.56	14.66 ± 8.93	1.31
Patients showing avoidance behaviour during the BAT	44 (37%)	39 (25%)	13 (22%)	6.13

*ASI* Anxiety Sensitivity Index, *BDI-II* Beck Depression Inventory-II, *CGI* Clinical Global Impression, *MI* Mobility Inventory, *PAS* Panic and Agoraphobia-Scale, *SIGH-A* Structured Interview Guide for the Hamilton Anxiety Scale. Due to missing values MI alone was available in 312 patients only (C/C: 108; C/T: 149; T/T: 55). CGI and SIGH-A scores were not available for one patient with C/C genotype. ASI score was not available for one patient with C/T genotype.

### Behavioural effects of neuron-specific deletion of *Rbfox1* in mice

**Fig. 3:**
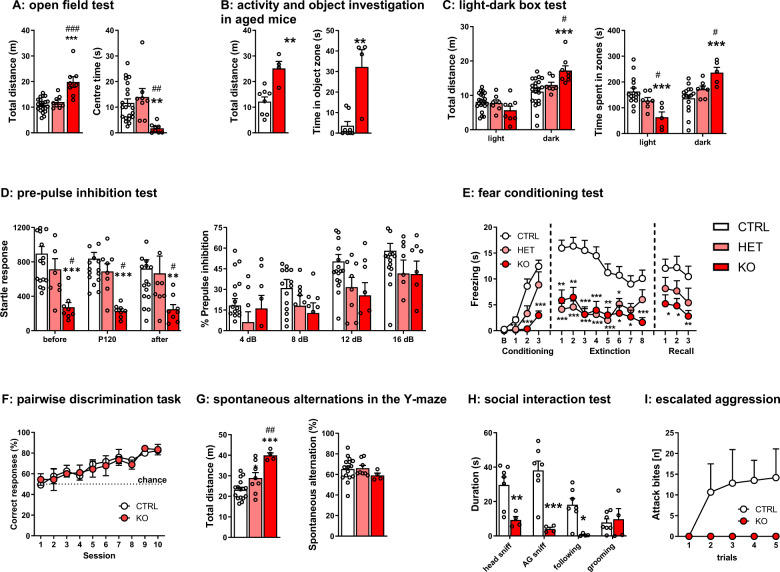
Effects of neuron-specific *Rbfox1* deletion on behavioural measures in male mice. **A** open field test: *Rbfox1-*KO mice displayed hyperactivity and reduced time in the centre in the open field test (CTRL: *n* = 21; HET: *n* = 8; KO: *n* = 8; **p < 0.01; *** *p* < 0.001 vs CTRL; ^##^*p* < 0.01; ^###^*p* < 0.001 vs HET; one-way ANOVA, Bonferroni test); **B** open field test and novel object exploration in 8-month-old mice: KO mice spent longer investigating a novel object placed into the open field (CTRL: *n* = 8; KO: *n* = 4; **p < 0.01 vs CTRL, Mann-Whitney test); **C** light-dark box test: KO mice again were hyperactive and spent more time in the dark zone (CTRL: *n* = 21; HET: *n* = 8; KO: *n* = 8; ***p < 0.001 vs CTRL; two-way ANOVA (genotype x zone), Bonferroni test); **D** pre-pulse inhibition test: KO mice had markedly reduced startle amplitude without changes in the sensorimotor gating (CTRL: *n* = 21; HET: *n* = 8; KO: *n* = 8; **p < 0.01; ***p < 0.001 vs CTRL; ^#^*p* < 0.05 vs HET; repeated measures ANOVA, Bonferroni test); **E** auditory fear conditioning and extinction: fear acquisition and extinction was impaired in the KO mice, and HET mice displayed impaired fear retention (CTRL: *n* = 21; HET: *n* = 8; KO: *n* = 8; *p < 0.05; **p < 0.01; ***p < 0.001 vs CTRL; repeated measures ANOVA, Bonferroni test); **F** touchscreen visual pairwise discrimination task: acquisition of the task was similar in CTRL and KO (CTRL: *n* = 5; KO: *n* = 4; repeated measures ANOVA); **G** spontaneous alternations in the Y-maze: the number of spontaneous alternations was not changed in KO (Kruskall-Wallis test) although the distance travelled during the test was significantly higher than CTRL (*n* = 8–16 per group; ***p < 0.001 vs CTRL; ^##^*p* < 0.01 vs HET; one-way ANOVA, Bonferroni test); **H** social interaction: KO spent significantly less time investigating unfamiliar stimulus mice (CTRL: *n* = 7; KO: *n* = 4; *p < 0.05; **p < 0.01; ***p < 0.001 vs CTRL; Welch’s t-tests); **I** escalated aggression paradigm: while aggressive behaviour increased during repeated sessions in CTRL, KO remained non-aggressive throughout testing (CTRL: *n* = 6; KO: *n* = 5; repeated measures ANOVA). Data is presented as means ± S.E.M.

Neuronal-specific deletion of *Rbfox1* resulted in a pronounced downregulation of *Rbfox1* relative to the neuronal marker *Synapsin 1* compared to more moderate downregulation relative to the housekeeping gene *Sdha* (i.e., also normalizing expression relative to all non-neuronal cells) without concomitant compensatory changes in either *Rbfox2* or *Rbfox3* (Supplementary Fig. 5A), and a reduction in body weight (Supplementary Fig. 5B). We observed persistent and pronounced hyperactivity in the KO mice in the open field and light-dark box (Fig. 3A–C), and marble burying tests (Supplementary Fig. 5C) compared to CTRL and HET. Interestingly, the hyperactivity was coupled with thigmotaxis, as the KO spent twice as much time as CTRL and HET moving adjacent to the maze walls (Supplementary Fig. 5D). This behaviour confounded the typical measures of anxiety in these tests. However, when a subset of the mice was tested again in the open field at the age of 8–9 months and was exposed to a novel object placed in the centre of the open field, the KOs spent three times longer than CTRL investigating it (Fig. 3B), suggesting an increase in the exploratory drive and the persistence of the hyperactive phenotype with age. In the pre-pulse inhibition test, conducted to assess the sensorimotor gating of startle response [46], KO mice showed a deficit in the acoustic startle response (Fig. 3D), but their ability to suppress their startle reflex when the startling stimulus was preceded by a sub-threshold pre-pulse stimulus (pre-pulse inhibition) was not impaired.

Next, we moved to more cognitively demanding tests. Here, we found that HET and KO mice had impairments in both fear acquisition and extinction in the auditory cued fear conditioning and extinction test (Fig. 3E). Compared to KO, HET mice were able to acquire fear conditioning like littermate controls but were unable to retain fear memory as evident from their reduced freezing in the extinction stage. Although it has been reported that some proposed rodent autism models show enhanced fear conditioning and impaired extinction [47], several transgenic mouse models for ASD display deficits in fear conditioning or no change from controls [48]. The deficit observed in our study was specific for cued fear learning, as neither associative learning in a touchscreen pairwise visual discrimination task (Fig. 3F; Supplementary Fig. 5E) nor spatial working memory (spontaneous alternations in the Y-maze) were impaired (Fig. 3G).

Finally, given the genetic association of *RBFOX1* with ASD and previous reports of a role in aggression, we assessed social interaction as well as male-male aggression. We observed significantly less social interest in the KO mice (Fig. 3H), which also manifested in a complete lack of aggressive behaviour (Fig. 3I). Thus, neuron-specific *Rbfox1* depletion in mice leads to hallmark features of ASD: repetitive-stereotyped and hyperactive locomotor behaviour, abnormalities in the fear circuitry, and impaired social interactions [49]. Such pronounced effects of neuron-specific loss of *Rbfox1* might thus also occur in human carriers of rare loss-of-function variants with high penetrance, underscoring the relevance of this gene for neurodevelopment.

## Discussion

The evidence from the genetic studies accrued here suggests that common genetic variation in *RBFOX1* goes along with a wide spectrum of psychiatric phenotypes, while rare CNVs in this gene contribute especially to ASD and SCZ, although this might be biased by the low number of studies investigating CNVs in other psychiatric disorders. The molecular-cellular effects of common genetic variation in *RBFOX1* are however yet elusive and likely include the regulation of gene expression. This may be operative only in certain cell types or developmental stages, as some of the major roles of RBFOX1 occur during early brain maturation [50], where it orchestrates downstream genetic networks implicated in neuronal development [51] via direct regulation of post-transcriptional programs. These gene networks are markedly inter-connected and enriched for genes relevant for cortical development and ASD [15] as well as MDD and SCZ susceptibility (Supplementary Table 3). On target transcripts, RBFOX1 regulates alternative splicing of tissue-specific exons [52] by binding to mRNA GCAUG motifs in the nucleus and affecting mRNA stability in the cytosol and thus has different roles in those intracellular compartments. Importantly, RBFOX1 promotes interneuron-specific connectivity in the developing neocortex [16] by regulating cell-type-specific splicing (parvalbumin [PV] vs. somatostatin [SST] interneurons). Loss of RBFOX1 in inhibitory interneurons causes significantly reduced synaptic transmission [53], by affecting membrane excitation and neurotransmission [54], resulting in reduced inhibition of the postsynaptic neuron and leading to excitatory/inhibitory (E/I) imbalance, a key feature of ASD. As PV + interneurons are regulators of E/I balance [55], this might link dysregulation of *RBFOX1* to E/I dysbalance and ASD susceptibility.

With respect to common genetic variation, *RBFOX1* is associated with all disorders combined, SCZ, MDD, and RT. Our neuroimaging data argue for an effect of *RBFOX1* genetic variation on the networks controlling emotional-associative learning, executive functioning, and emotional processing. Although our fMRI samples were relatively small and replications are necessary, we showed that rs6500744 risk genotype carriers display higher reactivity to emotional stimuli and reduced DLPFC activation during cognitive control, which are both linked to these mental disorders. Increased aggression found in C-allele carriers [14] is thus likely to be interpreted as reactive-impulsive, but not proactive, aggression. It must be considered that genetic variants in *RBFOX1* with small effect sizes in a polygenic scenario interact with many other variants to increase the risk towards mental disorders in a quasi-stochastic manner, probably explaining the broad psychopathological phenotype. In contrast, more penetrant CNVs with presumably stronger molecular effects may result in a more specific chronic neurodevelopmental behavioural syndrome.

While we cannot yet finally determine the functional consequences of *RBFOX1* genetic variation in humans, combining data from human and rodent experiments may suggest an increase in expression in MDD, anxiety, and (reactive) aggression. Up- and downregulation of *RBFOX1* however are likely to have different effects on the regulated gene networks [56], and human post-mortem data argues for reduced *RBFOX1* expression at least in ASD and SCZ. In line with this hypothesis, the remarkable behavioural phenotype of neuron-specific *Rbfox1* knockout mice suggests that loss-of-function of *RBFOX1* causes a behavioural syndrome characterised by hyperactivity, stereotypies, and specific cognitive and social impairments typical for ASD. A limitation of our study is that we only conducted behavioural experiments in male mice, but the findings will be extended to female animals in the future. As for the clinical phenotype in human *RBFOX1* CNV carriers, extending beyond “pure” ASD, we proposed that it is additionally shaped by genetic background and environmental factors. Given that our *Rbfox1* KO mouse line shows both high construct and face validity of ASD—as it is characterized by downregulation of *Rbfox1* in the brain and displays several ASD-related behaviours, we consider it an excellent animal model for ASD with an unprecedentedly robust behavioural phenotype.

Differential consequences of common and rare genetic variation, as we observe them for *RBFOX1*, may be a general principle in psychiatric genetics, where common variation in a gene might underly more generalized vulnerability, while rare, highly penetrant variation causes more specific phenotypes. In either case, it becomes clear that current diagnostic boundaries do not adequately reflect corresponding biological disease types. Given that approaches to modify *RBFOX1* expression are already at hand, which might be used in the sense of personalised mental health, this calls for mechanistic rather than theoretical, operationalised definitions of mental disorders.

## Supplementary information

Supplementary Methods

Supplementary Tables and Figures

Supplementary Tables 2, 4, and 6

Supplementary Video 1
