# Supplementary material for: Behavioural and functional evidence revealing the role of RBFOX1 variation in multiple psychiatric disorders and traits
Source: Mol Psychiatry. 2022 Aug 10;27(11):4464–73. doi: 10.1038/s41380-022-01722-4 (PMC9734045; doi:10.1038/s41380-022-01722-4)
Supplement: Supplementary file 1 — Supplementary Methods [file 41380_2022_1722_MOESM1_ESM.docx]

**MATERIALS AND METHODS**

**Common and rare genetic risk variants in *RBFOX1* in psychiatric phenotypes**

For SNP-based analysis of *RBFOX1* (NM_018723: chr16:6,069,132-7,763,340, GRCh37/hg19 UCSC RefSeq), we included a flanking region of 10 kb at 5’ and 5 kb at 3’ of the gene and retrieved information of all suggestive associated SNPs (p < 1e-05) from each summary statistics dataset. Gene-based association studies were performed on MAGMA v1.06 using the SNP-wise mean model without window around the gene, and the 1000 Genomes Project Phase 3 (European data only) as a reference panel. The enrichment of *RBFOX1* target genes, including those whose expression or splicing was regulated by RBFOX1, was assessed by a hypergeometric test. To investigate associations in multiple summary statistics available we used the tool PheWAS from the Atlas of GWAS Summary Statistics (https://atlas.ctglab.nl/).

**Flanker/Go-NoGo and Face matching tasks**

Task procedures*:* For the Face-matching task, participants in each trial viewed either a trio of angry or fearful faces or neutral geometric forms (see Figure 2A, left panel). They were instructed to select one of the two faces or forms presented at the bottom of the screen that was identical to the target stimulus presented at the top of the screen. In total, there were eight blocks with six images that were presented sequentially for 5 seconds, either three faces of each target affect (angry or fearful) and gender or six neutral forms. Task performance was assessed as a percentage of correctly answered trials for the face and form matching condition. For each participant, brain activation was estimated for each task condition by computing a general linear model using SPM8 including regressors for the face matching and the form matching blocks as well as six realignment parameters (3 translation, 3 rotation). We convolved a boxcar function representing the duration of blocks with the SPM8 canonical hemodynamic response function.

In each trial of the Flanker/Go-NoGo task, participants saw an array of five stimuli that included a central target arrow pointing left or right, flanked by two stimuli (arrows, boxes, or Xs, see Figure 2B, left panel) on either side. Participants were instructed to press a button corresponding to the direction of the central target arrow as fast and accurately as possible. In the “conflict monitoring” condition, the flanking arrows pointed either in the same direction (congruence, n=41 trials) or the opposite direction of the central arrow (incongruence, n=40 trials); the incongruence condition has been shown to slow down response times and indicates attentional capability to resolve conflict. In the “neutral“ condition (n=31 trials), the central arrow was flanked by boxes, measuring response execution without any conflict. In the “nogo” condition (n=33 trials), the central arrow was flanked by “Xs” which indicated that the participants had to withhold their response. The nogo condition is an established method to measure response inhibition. Each stimulus combination was presented for 800 milliseconds (ms) followed by a variable inter-trial interval of 2200–5200ms in which a fixation cross was shown in the centre of the screen. Task performance was examined by the accuracy for each condition (% correct) and the reaction time (RT in ms) for congruent, incongruent, and neutral conditions. All participants included in the current data analysis had an accuracy >60% for each condition. For each participant, brain activation was estimated for each task condition by computing a general linear model using SPM8 including regressors for the congruent, incongruent, nogo and neutral conditions as well as six realignment parameters (3 translation, 3 rotation). We convolved a stick function representing the onsets of trials with the SPM8 canonical hemodynamic response function.

**Fear conditioning and extinction**

Task procedure*:* The differential fear conditioning task during fMRI scanning consisted of three phases [Familiarisation (F) with 16 trials; acquisition (A) with 32 trials and extinction (E) with 16 trials of each CS+ and CS– (colored geometrical forms); presentation time: 2,000 ms with a variable inter-trial interval (ITI) of 4.785–7.250 s]. US, an aversive tone (100 ms white noise between 70 to 105 dB), was firstly familiarised (16 trials of isolated presentation), then pseudorandomly paired with one of the CSs (counterbalanced between subjects; partial reinforcement rate of 50 %) during acquisition, resulting in equal proportions of CS+_paired_ and CS+_unpaired_ trials. During the first level analysis using SPM5, we separated each phase into an early and a late part to account for temporal aspects. According to our operationalisation of simple fear learning and fear extinction, we compared CS+ in the late acquisition to CS+ in the late familiarisation and CS+ in the late extinction to CS+ in the late acquisition phase, respectively. Detailed information on the fMRI task, data acquisition, data quality control and analysis pathway using SPM is provided elsewhere ^1, 2^.

**Behavioural avoidance task (BAT)**

Task procedure: Briefly, after an anticipatory phase (sitting in front of the open test chamber) patients were asked to stay in a small, closed and dark test chamber for a maximum of 10 minutes (unknown for the patients). During BAT exposure the patients could refuse or end the test prematurely (passive and active avoidance behaviour, respectively) at any time. After BAT exposure a recovery phase followed (again sitting in front of the open test chamber). Reported fear was assessed immediately after each period (Likert scale ranging from 1 to 10). Heart rate was calculated from a continuously recorded electrocardiogram. Due to technical failures, heart rate was available only in a subsample of patients. To test for a significant association between rs6500744 genotype and BAT avoidance behaviour a chi-square test was conducted. The genotype effect on heart rate response was tested applying a mixed model of variance including genotype as a between-subject factor and BAT phase as within-subject factor.

**Animals**

**Quantitative PCR (qPCR)**

A total of 75 ng RNA per sample was reverse transcribed using the iScript™ cDNA Synthesis Kit (Bio-Rad), using both random hexamers and oligo(dT) primers. Target-specific quantitative PCR (qPCR) was performed as described before^3^ for *Actb, Hprt, B2m, Sdha* (as reference genes), *Syn1* (for normalization with a neuronal marker), *Rbfox1, Rbfox2*, and *Rbfox3* (as target genes) using target specific forward and reverse primers (final concentration: 0·3 µM each; see Supplementary Table 13 for primer sequences). Crossing point (Cp) values were calculated by the LightCycler 480 software (release 1.5.1.62) using the Second Derivative Maximum method. Relative gene expression levels were analysed using GenEx6 v3.1.3 (MultiD Analyses AB). For every target, a standard curve was created on the same plate as the target samples and Cp values were corrected for efficiencies calculated from these standard curves (see Supplementary Table 13). Missing data points were imputed.

**Mouse behavioural experiments**

*Open field test (OF) and novel object investigation.* The OF apparatus (Stoelting Europe, Dublin, Ireland) consisted of a 40 cm x 40 cm grey arena surrounded by black perspex walls (height: 35 cm) and a USB camera (The Imaging Source Europe, Bremen, Germany) was fixed on a metal arm above it. Each mouse was placed into the centre of the OF and allowed to explore freely for 3 min. The arena was virtually divided into a 15 cm x 15 cm centre area and a 12.5 cm wide periphery. Distance travelled and time spent in the centre was automatically quantified using ANY-maze automated tracking software (Stoelting Europe, Dublin, Ireland). Each mouse was initially tested in the OF at the age of 10-12 weeks. At the age of 8-9 months, a subset of the animals were re-assessed in the OF. After a 5-minute OF exposure, a small glass jar was placed into the centre of the OF, and visits to the object zone (ca 2 cm radius around the glass jar) were quantified using ANY-maze.

*Light-dark box (LDB).* The LDB apparatus (Stoelting Europe, Dublin, Ireland) consisted of an enclosure (W: 40 cm x L: 40 cm x H: 35 cm) divided equally into two 20 x 40 cm compartments: a brightly lit clear acrylic glass compartment (~400 lux) and a dark IR-transparent black perspex compartment (~3 lux) which were connected by an opening in the centre wall. Mice were individually placed into the light area and allowed to freely explore for 5 min. Distance travelled and time spent in each area, transitions between the compartments, and latencies to exit from and re-enter the light area were recorded using an IR-sensitive USB camera (The Imaging Source Europe, Bremen, Germany) and automatically tracked using ANY-maze software (Stoelting Europe, Dublin, Ireland).

*Touchscreen pairwise visual discrimination task.* Pairwise visual discrimination task was conducted in four operant chambers (Campden Instruments Ltd, Loughborough, UK) fitted with touchscreens that displayed the visual stimuli controlled by ABET II Software (Lafayette, IN, USA). The chambers were also equipped with liquid reward (strawberry milk, Müllermilch Erdbeer, Müller, Aretsried, Germany) dispensers, light and sound generators, and USB cameras, and were housed in sound-attenuated ventilated cubicles. Before the start of the experiments, mice were placed on mild food restriction to obtain a weight reduction of 5-10 % of their free-feeding weight. Pre-training consisted of several steps to gradually shape the required behaviours to perform the task (habituation to the test chambers, screen-touching, receiving the reward, initiating trials). Visual discrimination was performed as described previously ^4^. Briefly, the task consisted of pairwise discrimination of a rewarded (S+, “fan”) and unrewarded (S-, “marbles”) black and white images. The location of the S+ presentation was pseudo randomised. Touching the S+ triggered reward delivery while responding to the S- started a 5-s timeout with the house light on and no reward delivery. Sessions lasted until the animal completed 30 trials or after 60 min, whichever occurred earlier. The criterion for task acquisition was ≥ 80% accuracy (correct responses at S+) during the trial for two consecutive sessions.

*Spontaneous alternations in the Y-maze.* Spontaneous alternation was assessed in an apparatus (Stoelting Europe, Dublin, Ireland) which consisted of three identical arms (35 cm x 5 cm with 10 cm high walls) mounted in the shape of ‘Y’. Each mouse was placed into one of the arms and allowed to explore each of the three arms freely for 5 min. A spontaneous alternation occurred when a mouse visited different arms on each of the last three arm entries. The total number of arm entries and spontaneous alternations as well as total distance travelled in the Y-maze were recorded via a USB camera and quantified using ANY-maze. The percentage of spontaneous alternations of all arm entries was calculated according to the following formula:

$$Spontaneous alternation \left( \% \right)=100\times\frac{spontaneous alternations}{total arm entries-2}$$

*Pre-pulse inhibition (PPI) of the acoustic startle response (ASR).* PPI of the ASR was measured in the SR-LAB™ startle response system (San Diego Instruments, Inc., USA). Briefly, after 5 min acclimation to the background noise (65 dB white noise), mice were exposed to six startle pulse trials (120 dB broadband noise for 40 ms, 10 s inter-trial interval [ITI]). Then, mice were presented with 10 x no-stimulus, 10 x startle pulse, 10 x each prepulse (4, 8, 12, 16 dB above background = 69, 73, 77, 81 dB for 20 ms) followed after 80 ms by a startle pulse, 10 x prepulse only (81 dB) in pseudorandomized order with a variable ITI (20-30 s). The test session ended with six startle pulse trials separated by 10 s ITIs. The overall duration of this protocol was ca 35 min. The magnitude of the ASR (whole body reflex) to pulse only trials was averaged for each mouse and defined as startle amplitude. Percentage of PPI was calculated as described in Esen-Sehir et al. (2019) using the following formula:

$PPI\left( \% \right)=100\times\frac{{startle amplitude}_{startle trials}-{startle amplitude}_{prepulse+startle trials}}{{startle amplitude}_{startle trials}}$

*Cued fear conditioning and extinction.* Fear conditioning was conducted in the Ugo Basile fear conditioning system (Ugo Basile S.R.L, Gemonio, Italy) over three consecutive days. On day 1, for the acquisition of conditioned fear, mice were placed into a chamber with transparent walls and an electrified grid floor inside a sound-attenuated ventilated cubicle and allowed to habituate for 3 min. Fear conditioning was conducted with three pairings of a 30-s 80-dB 1000-Hz sound (conditioned stimulus, CS) terminating with a 2-s 0.4-mA scrambling footshock (inter-trial interval: 2 min). 24 hours later, on day 2, the conditioning chamber was transformed with black and white cardboard panels on the walls and a grey opaque perspex floor panel covering the electric grid. For the fear extinction test, mice were individually placed into the chamber and allowed to habituate for 3 min, followed by 16 30-s CS presentations with 5-s intervals. Another 24 hours later, fear extinction recall was assessed. The conditioning chamber was set up identically to that of the day before. Mice were individually placed into the chamber, allowed to habituate for 3 min, and exposed to three 30-s CS with 5-s intervals. As an index of conditioned fear, freezing behaviour (defined as inhibition of all movement except breathing) was recorded via a USB camera and automatically quantified using ANY-maze freezing detection module (threshold: 1000 ms).

*Resident-intruder test.* Aggressive behaviour was assessed using the escalated aggression paradigm of repeated daily resident-intruder tests. To increase the territoriality of the residents (the experimental animals), mice were pair-housed with an 8-week-old C57Bl/6J female mouse for one week before the aggression testing. The cages were not cleaned until after the aggression testing was finished. Before the daily testing, the female and any nesting material were removed from the cage. A juvenile intruder (5-week-old male C57Bl/6J) mouse was placed into the home cage of the resident and aggressive behaviour (attack latency and attack frequency) of the resident towards the intruder was manually scored for 5 min after the first attack. If no attack occurred within the first 5 min, the test was ended. Resident-intruder tests were conducted for five consecutive days.

*Social interaction test.* The social interaction test was conducted in clean home cages. Briefly, mice were individually placed into a clean cage and left to habituate for 15 min. Then, a social stimulus mouse (5-week-old male C57Bl/6J) was placed into the cage and the experimental mouse was allowed to investigate the stimulus mouse for 10 min. The duration of aspects of social and non-social behaviour: head sniffs, anogenital sniffs, chasing the stimulus mouse, and grooming were manually scored by an experienced experimenter.

*Marble burying.* 18 glass marbles (with a diameter of 15 mm) were evenly placed onto the clean bedding material in a clean cage. Each mouse was placed into the cage with marbles and allowed to explore for 30 min. Every 5 minutes, the number of buried marbles (more than 2/3 covered by bedding) was counted.

**Supplementary References:**

1. Kircher T, Arolt V, Jansen A, Pyka M, Reinhardt I, Kellermann T *et al.* Effect of cognitive-behavioral therapy on neural correlates of fear conditioning in panic disorder. *Biological psychiatry* 2013; **73**(1)**:** 93-101.

2. Lueken U, Straube B, Konrad C, Wittchen HU, Ströhle A, Wittmann A *et al.* Neural substrates of treatment response to cognitive-behavioral therapy in panic disorder with agoraphobia. *The American journal of psychiatry* 2013; **170**(11)**:** 1345-1355.

3. Freudenberg F, Candemir E, Chen X, Li LL, Esen-Sehir D, Schenk N *et al.* Hippocampal overexpression of NOS1AP promotes endophenotypes related to mental disorders. *EBioMedicine* 2021; **71:** 103565.

4. Horner AE, Heath CJ, Hvoslef-Eide M, Kent BA, Kim CH, Nilsson SRO *et al.* The touchscreen operant platform for testing learning and memory in rats and mice. *Nat Protoc* 2013; **8**(10)**:** 1961-1984.
