# Supplementary material for: Behavioural and functional evidence revealing the role of RBFOX1 variation in multiple psychiatric disorders and traits
Source: Mol Psychiatry. 2022 Aug 10;27(11):4464–73. doi: 10.1038/s41380-022-01722-4 (PMC9734045; doi:10.1038/s41380-022-01722-4)
Supplement: Supplementary file 2 — Supplementary Tables and Figures [file 41380_2022_1722_MOESM2_ESM.pdf]

Supplementary Tables

Supplementary Table 1. Description of samples used for analyses of common genetic variants (summary statistics from GWAS meta-analysis).

Supplementary Table 2. Common genetic risk variants (SNPs) in RBFOX1 gene showing suggestive associations with psychiatric conditions (Excel file)

Supplementary Table 3. Enrichment of *RBFOX1* target genes among the significant genes associated for each psychiatric condition

Supplementary Table 4. CNVs found in RBFOX1 in patients with psychiatric conditions (Excel file)

Supplementary Table 5. CNVs in *RBFOX1* identified in studies including cases and controls, frequencies and burden analyses.

Supplementary Table 6. Number of regulatory elements, H3K4me1 and H3K27ac peaks (data from ENCODE), detected in intronic CNVs in patients (Excel file).

Supplementary Table 7. Altered expression of *RBOX1* in brain regions of individuals with SCZ or ASD

Supplementary Table 8. Sample characteristics and behavioral performance for the Flanker/Go-nogo and face matching task (human imaging genetics sample)

Supplementary Table 9. Fear conditioning: Sociodemographic and psychological characteristics of panic disorder patients with C/C, C/T and T/T RBFOX1 rs6500744 SNP genotypes

Supplementary Table 10. Behavioural performance in the fear conditioning task in panic disorder patients

Supplementary Table 11. Neural correlates of rs6500744 SNP genotype differences in fear conditioning and extinction

Supplementary Table 12. Behavioral avoidance task: Mean heart rate responses (bpm) during phases of anticipation, exposure, and recovery of BAT assessment

Supplementary Table 13. Primer pairs used for qPCR

Supplementary Table 1. Description of samples used for analyses of common genetic variants (summary statistics from GWAS meta-analysis)

GWAS	Abbreviation	Participants	Reference
Attention-Deficit Hyperactivity Disorder	ADHD	19,099 Ca + 34,194 Co	Demontis et al., 2019
Aggression	AGG	87,485 individuals	Ip et al., 2019 (Biorxvs)
Autism Spectrum Disorder	ASD	18,382 Ca + 27,969 Co	Grove et al., 2019
Anorexia	ANO	16,992 Ca + 55,525 Co	Watson et al., 2019
Anxiety	ANX	12,655 Ca + 19,255 Co	Meier et al., 2019
Bipolar Disorder	BIP	20,352 Ca + 31,358 Co	Stahl et al., 2019
Major Depressive Disorder	MDD	135,458 Ca + 344,901 Co	Wray et al., 2018
Obsessive-Compulsive Disorder	OCD-MA	1,773 Ca + 6,122 Co + 915 trios	Arnold et al., 2018
Risk tolerance behavior	RT	975,353 individuals	Linner et al., 2019
Schizophrenia	SCZ	67,280 Ca + 86,912 Co	Schizophrenia Working Group of the Psychiatric Genomics Consortium, 2020
Tourette's Syndrome	TS	4,819 Ca + 9,488 Co	Yu et al., 2019
Cross-Disorder meta-analysis	CD-MA	232,964 Ca + 494,162 Co	Lee et al. 2019

Ca = Cases; Co = controls. Summary statistics from these GWAS meta-analyses were obtained from the Psychiatric Genomics Consortium (PGC; https://www.med.unc.edu/pgc/download-results/), Integrative Psychiatric Research Consortium (iPSYCH; https://ipsych.dk/en/research/downloads/), UK Biobank (https://www.ukbiobank.ac.uk/) and 23andMe (https://research.23andme.com/) or authors' request.

Supplementary table 3. Enrichment of *RBFOX1* target genes among the significant genes associated for each psychiatric condition

	ADHD	AGG	ANO	ANX	ASD	BIP	MDD	OCD	RT	TS	SCZ	CD
**Number of associated genes in the gene-based analysis***	20	3	41	2	13	1	262	1	279	2	438	266
**Number of *RBFOX1* target genes among the gene-based associated genes**	4	1	8	1	1	0	42	0	46	1	60	42
**p-value of the hypergeometric test**	0.140	0.287	0.069	0.219	0.327	N/A	**0.016**	N/A	**0.010**	0.219	**0.042**	**0.019**

* Number of genes associated in the GWAS of each psychiatric condition overcoming gene-wide significance (Bonferroni correction; p = 20000/0.05 = 2.5E-06). ADHD, attention-deficit and hyperactivity disorder; AGG, childhood aggression; ANO, anorexia nervosa; ANX, anxiety; ASB, antisocial behaviour; ASD, autism spectrum disorder; BP, bipolar disorder; CD, cross-disorder meta-analysis; MDD, major depressive disorder; OCD, obsessive-compulsive disorder; SCZ, schizophrenia; RT, risk tolerance; TS, Tourette syndrome. Total number of genes in the genome: 20000 genes; 2499 *RBFOX1* target genes (Lee et al. 2016).

Supplementary Table 5. CNVs in *RBFOX1* identified in studies including cases and controls, frequencies and burden analyses.

Study (PMID)	Disorder	Total number of cases	Total number of controls	Cases with *RBFOX1* CN gain	Freq. RBFOX1 CN gain in cases	Controls with *RBFOX1* CN gain	Freq *RBFOX1* CN gain in controls	Cases with *RBFOX1* CN loss	Freq. *RBFOX1* CN loss in cases	Controls with *RBFOX1* CN loss	Freq. *RBFOX1* CN loss in controls	Ratio *RBFOX1* CNVs in cases vs controls	Burden Test all CNVs	Burden Test CN gain	Burden Test CN loss
Elia et al., 2010 (19546859)	ADHD	335	2026	2	0.60%	0	0.00%	1	0.30%	0	0.00%	-	<u>0.004</u>	0.151	<u>0.019</u>
Lionel et al.. 2011 (21832240)	ADHD	248	2357	0	0.00%	0	0.00%	1	0.40%	0	0.00%	-	0.096	-	0.096
Jarik et al., 2014 (23164820)	ADHD	489	1285	1	0.20%	8	0.62%	3	0.61%	4	0.31%	0.9 : 1	1	1	0.296
**TOTAL**	**ADHD**	**1072**	**5668**	**3**	**0.28%**	**8**	**0.14%**	**5**	**0.47%**	**4**	**0.07%**	**3.5 : 1**	-	-	-
Prasad et al., 2012 (23275889)	ASD	676	5139	0	0.00%	0	0.00%	3	0.44%	0	0.00%	-	<u>0.001</u>	-	<u>0.002</u>
Bacchelli et al., 2020 (32081867)	ASD	128	363	0	0.00%	0	0.00%	2	1.56%	2	0.55%	2.8 : 1	0.278	-	0.287
Sebat et al., 2007 (17363630); Martin et al., 2007 (17503474)	ASD	195	196	0	0.00%	0	0.00%	1	0.51%	0	0.00%	-	0.497	-	0.495
Griswold et al., 2012 (22543975)	ASD	813	592	0	0.00%	0	0.00%	4	0.49%	0	0.00%	-	0.108	-	0.109
Girirajan et al., 2013 (23375656)	ASD	2588	580	0	0.00%	0	0.00%	6	0.23%	0	0.00%	-	0.298	-	0.298
Kushima et al., 2018 (30208311)	ASD	1108	2095	0	0.00%	0	0.00%	2	0.18%	3	0.14%	1.3 : 1	0.552	-	0.568
Kanduri et al., 2016 (26052927)	ASD	80	269	1	1.25%	0	0.00%	0	0.00%	2	0.74%	1.7 : 1	0.542	-	1
Chen et al., 2017 (28931914)	ASD	335	1093	1	0.30%	0	0.00%	0	0.00%	0	0.00%	-	0.231	0.234	-
**TOTAL**	**ASD**	**5923**	**10327**	**2**	**0.03%**	**0**	**0.00%**	**18**	**0.30%**	**7**	**0.07%**	**5.0 : 1**	-	-	-
Grozeva et al 2010 (20368508)	BIP	1697	2806	2	0.12%	0	0.00%	0	0.00%	0	0.00%		0.142	0.150	-
**TOTAL**	**BIP**	**1697**	**2806**	**2**	**0.12%**	**0**	**0.00%**	**0**	**0.00%**	**0**	**0.00%**	**-**	-	-	-

Grünblatt et al., 2017 (29179725)	OCD	121	124	0	0.00%	0	0.00%	1	0.83%	0	0.00%	-	0.503	-	0.498
**TOTAL**	**OCD**	**121**	**124**	**0**	**0.00%**	**0**	**0.00%**	**1**	**0.83%**	**0**	**0.00%**	**-**	-	-	-
Kushima et al., 2018 (30208311)	SCZ	2458	2095	0	0.00%	0	0.00%	6	0.24%	3	0.14%	1.7 : 1	0.340	-	0.333
Kushima et al., 2017 (27240532)	SCZ	1699	824	0	0.00%	0	0.00%	3	0.18%	1	0.12%	1.4 : 1	0.610	-	0.607
Costain et al., 2013 (23813976)	SCZ	454	416	0	0.00%	0	0.00%	7	1.54%	5	1.20%	1.3 : 1	0.444	-	0.452
**TOTAL**	**SCZ**	**4611**	**3335**	**0**	**0.00%**	**0**	**0.00%**	**16**	**0.35%**	**9**	**0.27%**	**1.3 : 1**	-	-	-
Huang et al., 2018 (28641109)	Tourette syndrome	2434	4093	1	0.04%	1	0.02%	6	0.25%	7	0.17%	1.5 : 1	0.304	0.601	0.344
Fernandez et al., 2012 (22169095)	Tourette syndrome	460	1131	1	0.22%	3	0.27%	1	0.22%	2	0.18%	1.0 : 1	1	1	0.642
McGrath et al., 2015 (25062598)	Tourette syndrome	1086	1789	1	0.09%	0	0.00%	0	0.00%	0	0.00%	1	0.370	0.387	-
**TOTAL**	**Tourette syndrome**	**3980**	**7013**	**3**	**0.08%**	**4**	**0.06%**	**7**	**0.18%**	**9**	**0.13%**	**1.4 : 1**	-	-	-
**TOTAL**	**ALL**	**17404**	**29273**	**10**	**0.06%**	**12**	**0.04%**	**47**	**0.27%**	**29**	**0.10%**	**2.3 : 1**	-	-	-

This table includes only 18 studies out of 34 (from Supplementary Table 4) for which information about CNVs in controls was available. ADHD, attention deficit hyperactivity disorder; ASD, autism spectrum disorder; BIP, bipolar disorder; CN, copy number; CNVs, copy number variants; Freq., frequency; OCD, obsessive-compulsive disorder; PMID, PubMed ID; SCZ, schizophrenia. Burden analysis performed using PLINK v.1.07. Underlined values correspond to p-values <0.05.

Supplementary Table 7. Altered expression of *RBOX1* in brain regions of individuals with SCZ or ASD

**Gene Symbol / Transcript symbol**	**Disorder**	**FC**	***p*-value**	**FDR**	**Probe**	**Brain region (*post mortem*)**	**Study (PMID) or GEO ID**	**Sample, cases vs. controls**
RBFOX1	ASD	-1.20	7.11E-03	**0.068**	N/A	frontal and temporal cortex	Parikshak et al., 2016 (27919067)	48 ASD vs. 49 control
RBFOX1	ASD	-1.41	2.20E-02	N/A	ILMN_1814316	temporal cortex	Schwede et al., 2018 (29859039)	15 ASD vs. 16 control
RBFOX1	ASD	-1.40	3.12E-02	N/A	ILMN_2359168	temporal cortex	Schwede et al., 2018 (29859039)	15 ASD vs. 16 control
RBFOX1	ASD	-1.39	1.59E-02	N/A	ILMN_1731507	temporal cortex	Schwede et al., 2018 (29859039)	15 ASD vs. 16 control
RBFOX1	ASD	-1.32	N/A	**0.043**	ILMN_1731507	prefrontal and temporal cortex	Schwede et al., 2018 (29859039)	15 ASD vs. 16 control
RBFOX1	ASD	-1.10	9.07E-04	**0.025**	N/A	frontal and temporal cortex	Gandal et al., 2018 (30545856)	51 ASD vs. 936 control
RBFOX1-002	ASD	-1.14	8.69E-06	**0.006**	N/A	frontal and temporal cortex	Gandal et al., 2018 (30545856)	51 ASD vs. 936 control
RBFOX1-017	ASD	1.17	3.06E-02	0.369	N/A	frontal and temporal cortex	Gandal et al., 2018 (30545856)	51 ASD vs. 936 control
RBFOX1-016	ASD	1.35	2.26E-02	0.325	N/A	frontal and temporal cortex	Gandal et al., 2018 (30545856)	51 ASD vs. 936 control
RBFOX1-002	SCZ	-1.02	1.55E-02	0.170	N/A	frontal and temporal cortex	Gandal et al., 2018 (30545856)	559 SCZ vs. 936 control
RBFOX1	SCZ	1.10	3.47E-02	0.278	221217_s_at	prefrontal cortex	GSE21138	30 SCZ vs. 29 control
RBFOX1	SCZ	-1.26	4.89E-02	0.328	1553422_s_at	striatum	GSE53987	18 SCZ vs. 18 control
RBFOX1	SCZ	-1.17	4.60E-04	**0.094**	221217_s_at	prefrontal cortex	GSE53987	15 SCZ vs. 19 control
RBFOX1	SCZ	-1.18	2.38E-02	0.129	221217_s_at	hippocampus	GSE53987	15 SCZ vs. 18 control
RBFOX1	SCZ	-1.04	4.33E-02	0.327	7993083	cerebellum	GSE35978	44 SCZ vs. 50 control
RBFOX1	SCZ	-1.04	4.33E-02	0.327	7993083	cerebellum	GSE35974	44 SCZ vs. 50 control

N/A, data not available; FC, fold change; FDR, False Discovery Rate; SCZ, schizophrenia; ASD, autism spectrum disorder. In bold, overcoming multiple testing corrections at 10% FDR.

Supplementary Table 8. Sample characteristics and behavioral performance for the Flanker/Go-nogo and face matching task (human imaging genetics sample)

	Flanker/Go-Nogo (n=324)			Face matching (n=313)		
	C-carrier	T/T carrier	Test-statistic	C-carrier	T/T carrier	Test-statistic
Demographics						
Age, mean +/- SD	32,9 ±9,7	33,0 ±10,1	*t*(322)=0,100, *p*=0,920	33,7 ±9,8	33,5 ±10,04	*t*(311)=-0,111, *p*=0,912
Sex (males/females)	119/134	36/35	X^2=0,299, p=0,584	114/129	36/34	X^2=0,444, p=0,505
Education (years), mean +/- SD	15,4 ±2,4	15,1 ±2,6	*t*(322)=-0,960, *p*=0,338	15,5 ±2,5	15,3 ±2,7	*t*(311)=-0,640, *p*=0,523
Site (Berlin/Mannheim/ Bonn)	72/80/101	16/24/31	X^2=0,989, p=0,610	73/69/101	16/24/30	X^2=1,643, *p*=0,440
Handedness (right/left/both)	230/16/6	62/8/1	n/a*	220/16/6	61/8/1	n/a*
fMRI task performance						
Incongruent (% correct), mean ±SD	97,85 ±3,76	97,36 ±4,14	F(1,317)=1,089, *p*=0,30[a,d]			
Congruent (% correct), mean ±SD	99,42 ±2,44	98,42 ±4,68	**F(1,317)=5,74, *p*=0,017**			
Neutral (% correct), mean ±SD	99,07 ±3,12	98,73 ±4,51	F(1,317)=0,54, *p*=0,46			
Nogo (% correct, no response), mean ±SD	91,65 ±6,4	91,55 ±5,75	F(1,317)=0,02, *p*=0,90			
Incongruent, RT (ms), mean ±SD	632,78 ±117,07	623,60 ±96,71	F(1,317)=0,62, *p*=0,41[a,b,c]			
Congruent, RT (ms), mean ±SD	576,19 ±112,00	571,44 ±94,93	F(1,317)=0,23, *p*=0,63[a,b,c]			
Neutral, RT (ms), mean ±SD	608,33 ±113,65	597,40 ±94,94	F(1,317)=0,87, *p*=0,35[a,b,c]			
Faces (% correct)				98,58 ±3,46	98,45 ±3,11	F(1,306)=0,02, *p*=0,90
Forms (% correct)				97,39 ±3,93	95,89 ±4,68	**F(1,306)=7,04, *p*=0,008[b]**

Type of statistical test for group comparisons: Normally distributed variables → independent t-tests or univariate ANOVAs including genotype and sex (between-subjects factors), and age and site (covariates of no interest), dichotomous variables → χ^2 test. Besides genotype effects univariate ANOVAs showed significant effects of [a]sex, [b]age, [c]site, [d]genotype x sex interaction. [#]statistical test not possible due to low number of “both” handedness. *Please note*: We controlled all second-level SPM12 analyses for the effects of sex, age, site, and behavioral covariates corresponding to the fMRI contrast of interest (see methods for details). Abbreviations: ms = milliseconds, RT = reaction time, SD = standard deviation.

Supplementary Table 9. Fear conditioning: Sociodemographic and psychological characteristics of panic disorder patients with C/C, C/T and T/T RBFOX1 rs6500744 SNP genotypes

	**C/C (n=15)**	**C/T (n=21)**	**T/T (n=11)**	***F/Chi²***	***Post-hoc tests***
Age in years	40.67±8.00	38.14±10.70	31.45±9.77	2.98	
Female gender	11 (73%)	15 (71%)	7 (64%)	0.31	
Years of education				1.05	
≤ 8	1	2	1		
9 – 11	8	11	4		
≥ 12	6	8	6		
Study Center				1.20	
Center 1	4	7	4		
Center 2	1	1	0		
Center 3	3	5	2		
Center 4	7	8	5		
Digit span forward	7.60±2.20	7.95±1.69	7.45±2.77	0.23	
Digit span backward	7.00±2.10	7.00±2.05	7.18±2.04	0.03	
TMT-A	25.60±5.17	27.50±9.99	21.54±4.12	2.22	
TMT-B	58.27±18.40	56.29±18.71	54.18±16.41	0.16	
CGI	5.53±0.74	5.43±0.60	5.36±0.51	0.25	
SIGH-A	25.67±6.02	23.33±4.48	24.09±5.67	0.86	
MI alone	2.90±0.91	2.34±1.22	2.45±0.91	1.29	
PAS	29.83±7.35	24.61±8.62	27.18±10.95	1.53	
ASI	37.00±7.31	27.95±9.64	29.45±10.02	4.63*	C/C>C/T
BDI-II	23.67±9.08	14.48±4.91	12.91±7.85	9.62***	C/C>C/T, C/C>T/T

ASI: Anxiety Sensitivity Index; BDI-II: Beck Depression Inventory-II; CGI: Clinical Global Impression; MI: Mobility Inventory; PAS: Panic and Agoraphobia-Scale; SIGH-A: Structured Interview Guide for the Hamilton Anxiety Scale; TMT: Trail Making Test; *: $p < 0.05$; ***: $p < 0.001$

Supplementary Table 10. Behavioural performance in the fear conditioning task in panic disorder patients

	C/C (n=19)		T/C (n=34)		T/T (n=12)	
	Mean	SD	Mean	SD	Mean	SD
**Aversiveness US**	8,00	1,15	7,91	1,74	7,08	2,99
**Fam.: Valence CS+**	3,16	0,83	3,09	1,08	3,00	0,95
**Fam.: Arousal CS+**	2,21	0,71	2,09	1,08	2,58	1,00
**Fam.: Valence CS-**	3,11	0,94	2,94	1,28	3,42	1,08
**Fam.: Arousal CS-**	2,21	0,85	2,03	1,14	2,25	1,06
**Acq.: Valence CS+**	3,05	1,08	2,94	1,18	2,92	0,90
**Acq.: Arousal CS+**	2,37	0,90	2,21	1,25	2,25	0,97
**Acq.: Valence CS-**	3,16	1,01	3,00	1,39	3,42	1,00
**Acq.: Arousal CS-**	2,16	1,12	1,97	1,14	2,17	1,03
**Ext.: Valence CS+**	3,53	0,77	2,94	1,15	3,25	0,87
**Ext.: Arousal CS+**	1,95	0,91	1,94	1,07	1,92	1,38
**Ext.: Valence CS-**	3,42	0,96	3,06	1,25	3,50	1,09
**Ext.: Arousal CS-**	2,16	1,07	1,91	1,19	1,92	1,24

Acq.: acquisition phase; CS: conditioned stimulus; Ext.: extinction phase; Fam.: familiarization phase; US: unconditioned stimulus.

Supplementary Table 11. Neural correlates of rs6500744 SNP genotype differences in fear conditioning and extinction

Anatomical region	Cluster extension	BA	Coordinates *x*	*y*	*z*	*t*-value	no. voxels
C/C >T/T in simple fear conditioning (Contrast: CS+ in late familiarization < CS+ in late acquisition)							
Right MFG	Bilateral dmPFC, dACC, SFG, right IFG	9,10,24,32,46	44	50	20	4.80	3931
Right MTG	Right STG, insula, precentral gyrus, rolandic operculum, SMG	21,22,39,41	34	-16	42	4.34	6093
Occipital visual cortex		17,18	-36	-88	12	4.29	791
Left postcentral gyrus	Left STG, MTG, IPL	3,39,41,42	-50	-18	52	4.18	2604
Left Insula	Left thalamus, MTG, putamen		8	-30	-14	4.17	2219
Right lingual gyrus	Right calcarine gyrus, fusiform gyrus	17,18,19	18	-84	4	4.14	1539
Precuneus	PCC, paracentral lobule	31	-8	-48	42	3.71	1150
Left precentral gyrus		9	-44	14	32	3.60	306
Left Amygdala (ROI)			-26	-2	-14	2.83	20
C/C >T/T in differential fear conditioning (Contrast: CS+ in late acquisition > CS- in late acquisition)							
PCC	Precuneus	7,31	-14	-50	40	4.32	1436
Right angular gyurs	Right STG, MTG	22,40	48	-58	40	3.90	852
dmPFC	SMA	8	10	24	48	3.74	298
Left angular gyrus	Left IPL	40	-52	-62	44	3.69	464
Lingual gyrus	Calcarine gyrus	18	10	-78	0	3.49	478
STG		22	-48	-42	14	3.27	185
Cuneus		19	16	-80	30	3.04	157
C/C >T/T in fear extinction (Contrast: CS+ in late acquisition > CS+ in late extinction)							
dmPFC	dACC, SFG,	6,8,9,32	4	66	14	4.47	3207
Precentral gyrus		6	-36	-2	44	3.55	261
Right IFG	MFG	45	38	22	24	3.30	637

Participants are patients with panic disorder. Neuroimage Coordinates are listed in MNI space. ROI: region of interest analysis was performed within bilateral amygdala. Significance level: uncorrected $p < 0.005$, cluster with at least 141 voxels. BA: brodmann areas; dACC; Dorsal anterior cingulate cortex; dmPFC: dorsomedial prefrontal cortex; IFG: Inferior frontal gyrus; IPL: Inferior parietal lobule; MFG: Middle frontal gyrus; MTG: Middle temporal gyrus; PCC; Posterior cingulate cortex; SFG: Superior frontal gyrus; SMA: Supplementary motor cortex; STG: Superior temporal gyrus.

Supplementary Table 12. Behavioral avoidance task: Mean heart rate responses (bpm) during phases of anticipation, exposure, and recovery of BAT assessment, respectively, and heart rate increase from last minute of anticipation phase to first minute of exposure phase (Δbpm) in panic disorder patients with C/C, C/T and T/T RBFOX1 rs6500744 SNP genotypes showing no avoidance behavior and reporting at least moderate fear during the task.

	**C/C (n=34)**	**C/T (n=54)**	**T/T (n=18)**
Anticipation	80.39±12.52	78.09±11.89	76.23±4.44
Exposure	81.45±12.86	79.37±12.97	77.44±5.24
Recovery	77.35±10.28	76.32±11.05	77.19±5.25
Initial response during BAT exposure	5.42±11.44	2.25±9.06	-0.66±5.82

BAT: behavioral avoidance task; bpm: beats per minute.

Supplementary Table 13. Primer pairs used for qPCR

Name (Target)[a]	Sequences[b]	Target transcripts[c]	Std. curve[d]
Actb (Mus musculus actin, beta)	F: GCTTCTTTGCAGCTCCTTCG<br>R: ATGCCGGAGCCGTTGTC	60-156 of NM_007393.5	y = -4.04x+35.3<br>r^2 = 0.997<br>E = 0.767
B2m (Mus musculus beta-2 microglobulin	F: ACTGACCGGCCTGTATGCTA<br>R: CAATGTGAGGCGGGTGGAA	93-217 of NM_009735.3	y = -3.29x+36.9<br>r^2 = 0.955<br>E = 1.013
Hprt (Mus musculus hypoxanthine guanine phosphoribosyl transferase)	F: TGCTGACCTGCTGGATTACA<br>R: TTTATGTCCCCCGTTGACTGA	371-490 of NM_013556.2	y = -3.91x+37.6<br>r^2 = 0.996<br>E = 0.803
Rbfox1 (Mus musculus RNA binding protein, fox-1 homolog (C. elegans))	F: AGACTGTCAACCCCTACACCA<br>R: CATAGAAGTCGGGGCTGTAGAC	Amplification of all 9 verified and 44 predicted transcripts with 75 bp amplicon size	y = -4.06x+38.0<br>r^2 = 0.970<br>E = 0.764
Rbfox2 (Mus musculus RNA binding protein, fox-1 homolog (C. elegans))	F: GGGATGCAGAACGAGCCAC<br>R: CTGAACCATTGCGTCAGGAG	Amplification of 3/12 verified transcripts (NM_053104.6, NM_175387.3, NM_001110827.2) and 12/43 predicted transcripts with 102 bp amplicon size	y = -5.09x+41.5<br>r^2 = 0.959<br>E = 0.572
Rbfox3(Mus musculus RNA binding protein, fox-1 homolog (C. elegans))	F: ATCGTAGAGGGACGGAAAATTGA<br>R: GTTCCCAGGCTTCTTATTGGTC	Amplification of 5/6 verified transcripts (NM_001039167.1, NM_001039168.1, NM_001024931.2, NM_001285436.1, NM_001285438.1) 22/22 predicted transcript with 72 bp amplicon size	y = -4.32x+38.7<br>r^2 = 0.991<br>E = 0.703
Sdha (Mus musculus succinate dehydrogenase complex, subunit A, flavoprotein (Fp))	F: GGACAGGCCACTCACTCTTAC<br>R: CACAGTGCAATGACACCACG	616-745 of NM_023281.1	y = -3.30x+36.7<br>r^2 = 0.940<br>E = 1.01
Syn1 (Mus musculus synapsin I)	F: TGCCCAGATGGTTCGACTAC<br>R: CACAGGGTATGTTGTGCTGC	816-927 of NM_013680.4 and NM_001110780.1	y = -4.83x+41.8<br>r^2 = 0.950<br>E = 0.611

[a] Name of the primer pair/target gene and full name of the target gene (protein names that differ from the gene name are written in square brackets).
[b] Nucleotide sequences of the forward (F) and reverse (R) primers.
[c] Region of the target gene amplified by the primer pair.
[d] Linear function and coefficient of determination (r^2) of the standard curve and efficiency (E) of the primer calculated from the slope.

Supplementary Figures:
Supplementary Figure 1. Copy-number variations in *RBFOX1* in controls and patients with other psychiatric disorders than ASD and SCZ.
Supplementary Figure 2. Distribution of non-coding copy number variants (CNVs) in *RBFOX1* in patients with psychiatric conditions and overlap with transcriptional regulatory elements.
Supplementary Figure 3. *RBFOX1* expression in different tissues from human samples.
Supplementary Figure 4. RBFOX1 gene expression in different human brain regions.
Supplementary Figure 5. Effects of neuron-specific deletion of *Rbfox1* on body weight, gene expression, and behaviour in mice.
Supplementary Figure 6. Schematic representation of the cohorts and experimental schedule used in the mouse experiments.

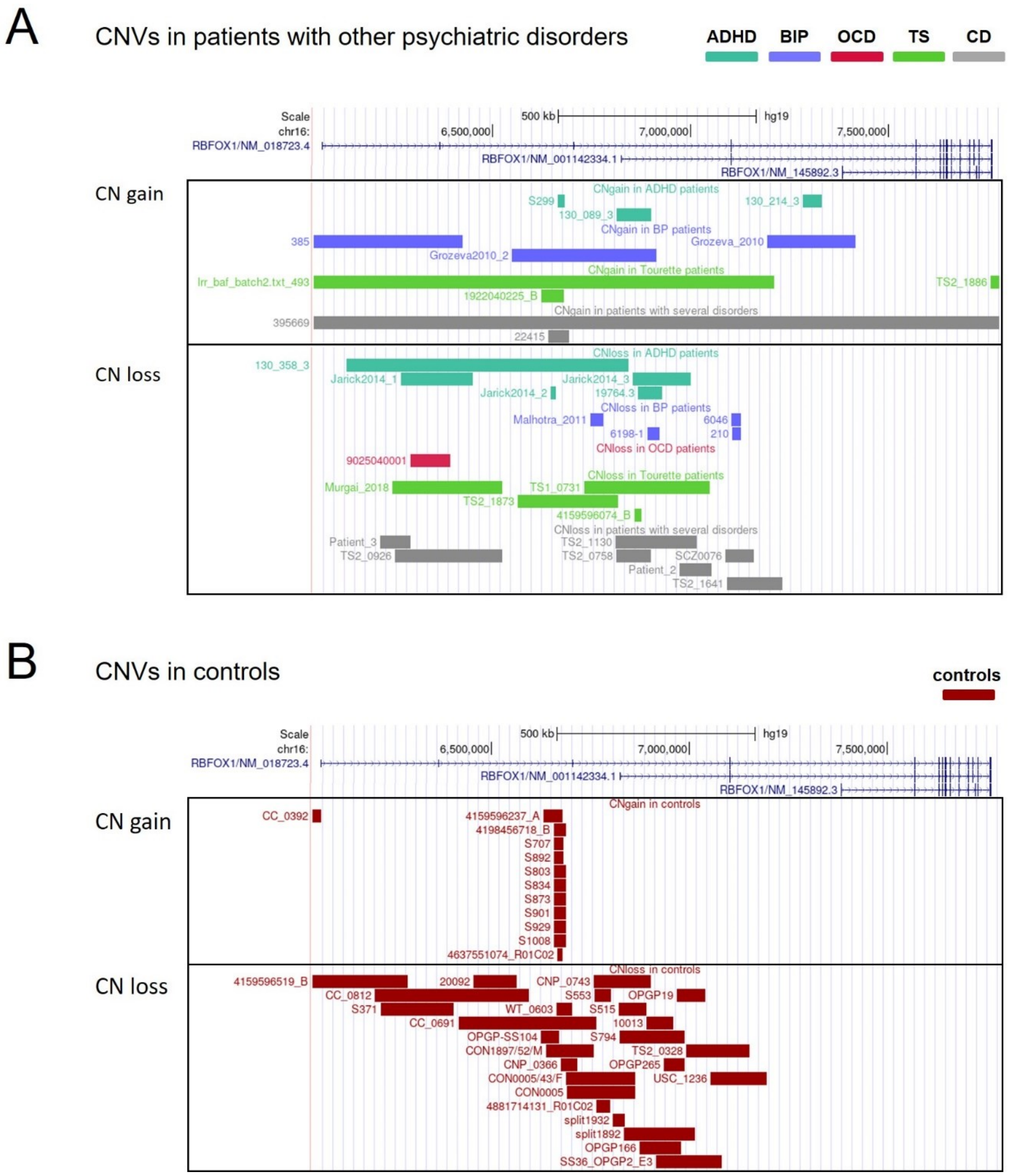

Supplementary Figure 1. Copy number variants in *RBFOX1* identified in A) patients with other psychiatric disorders, B) controls described in the 18 papers used for the burden test analysis.

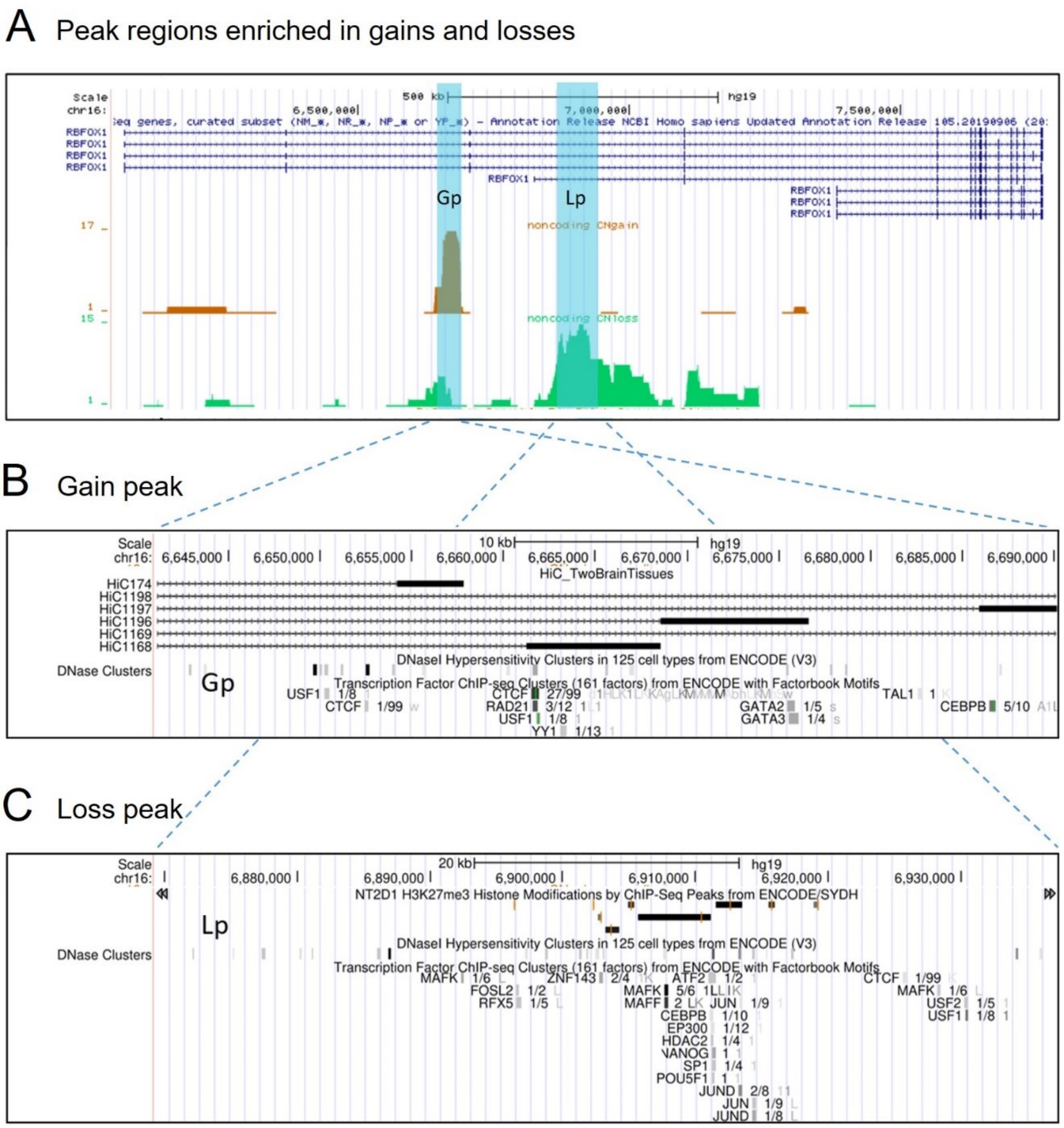

Supplementary Figure 2. Distribution of non-coding copy number variants (CNVs) in *RBFOX1* in patients with psychiatric conditions and overlap with transcriptional regulatory elements. a) Distribution of non-coding CNVs in *RBFOX1* in patients. Peak regions enriched in CNVs are highlighted in blue. Gp, gain peak, corresponds to the region where more CN gains are concentrated; Lp, loss peak, corresponds to the region where more CN losses are concentrated. b) Distribution of ChIP-seq signatures from ENCODE present in the Lp region. c) Distribution of ChIP-seq signatures from ENCODE present in the Gp region.

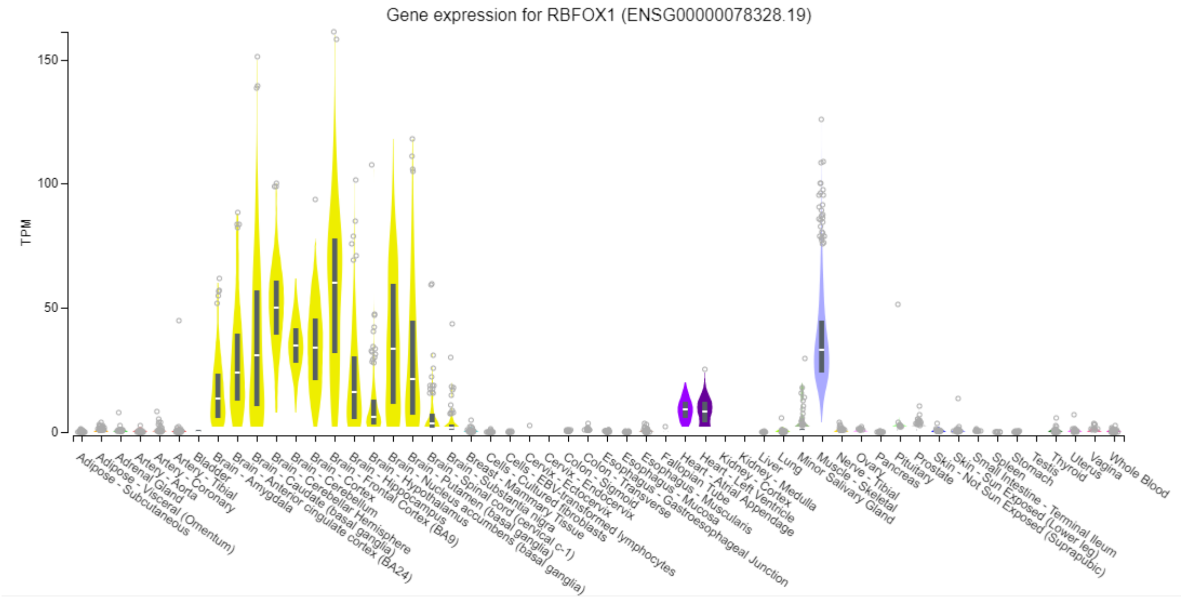

Supplementary Figure 3. *RBFOX1* expression in different tissues from human samples (GTEx database).

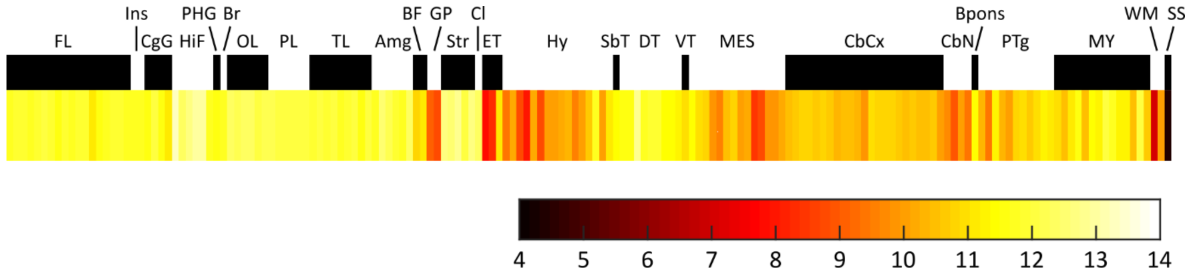

Supplementary Figure 4. *RBFOX1* gene expression in different brain regions of the human brain (FL, frontal lobe; Ins, Insula; CgG, Cingulate Gyrus; HiF, Hippocampal Formation; PHG, parahippocampal gyrus; Br, piriform cortex; OL, occipital lobe; PL, parietal lobe; TL, temporal lobe; Amg, amygdala; BF, basal forebrain; GP, globus pallidus; Str, striatum; Cl, claustrum; ET, epithalamus; Hy, hypothalamus; SbT, subthalamus; DT, dorsal thalamus; VT, ventral thalamus; MES, mesencephalon; CbCx, cerebellar cortex; CbN, cerebellar nuclei; Bpons, basal part of pons; PTg, pontine tegmentum; MY, myencephalon; WM, white matter; SS, sulci & spaces). Data was obtained from the Allen Human Brain Atlas (http://human.brain-map.org/) (Hawrylycz et al. 2012) and depicted as the average from two different probes.

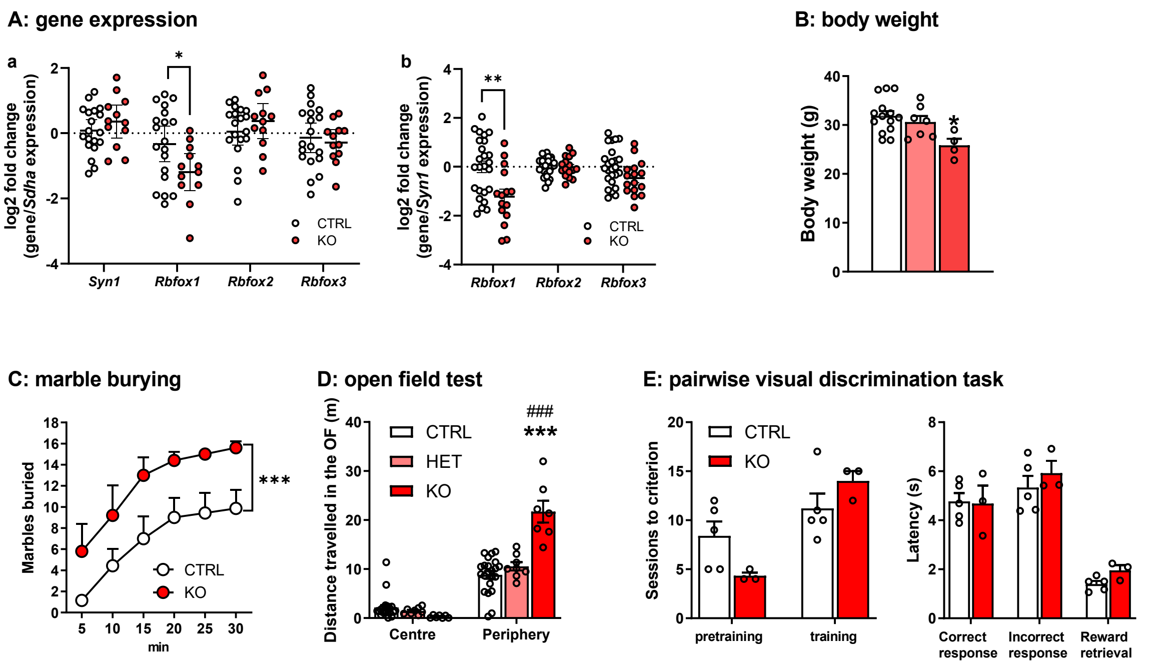

Supplementary Figure 5. Effects of neuron-specific deletion of *Rbfox1* on body weight, gene expression, and behaviour in mice. **A,** neuron-specific deletion of *Rbfox1* resulted in selective brain-wide *Rbfox1* downregulation relative to the housekeeping gene *Sdha* (a), and even more pronounced downregulation relative to the neuronal marker *Synapsin1 (Syn1)* (b), while *Rbfox2 and Rbfox3* expression remained unaltered (n=12-18 tissue samples per group; * - p<0.05; ** - p<0.01 vs CTRL; two-tailed t-tests). **B,** *Rbfox1*-KO had lower body weight than CTRL and HET at 10-14 weeks (n=4-14 per group; * - p<0.05 vs CTRL; one-way ANOVA; Fisher's LSD test). **C,** *Rbfox1*-KO buried more marbles than CTRL in the marble-burying test (n=5-7 per group; * - p<0.05 vs CTRL; Friedman's test), but this appeared to be due to excessive digging and displacement of bedding rather than anxiety (Supplementary Video 1). **D,** *Rbfox1*-KO displayed marked thigmotaxis in the open field test compared to CTRL and HET, moving mainly in the peripheral area of the open field (n=7-21 per group; *** - p<0.001 vs CTRL; ### - p<0.001 vs HET; two-way ANOVA; Bonferroni post hoc test). **E,** there were no differences between CTRL and KO in the acquisition of the touchscreen pairwise discrimination task (as measured by the number of sessions completed before reaching the criterion); additionally, similar response latencies to stimuli and reward retrieval suggest that CTRL and KO did not differ in motivation to perform the task (n=4-5 per group; two-tailed t-tests). Data is presented as means ± SEM.

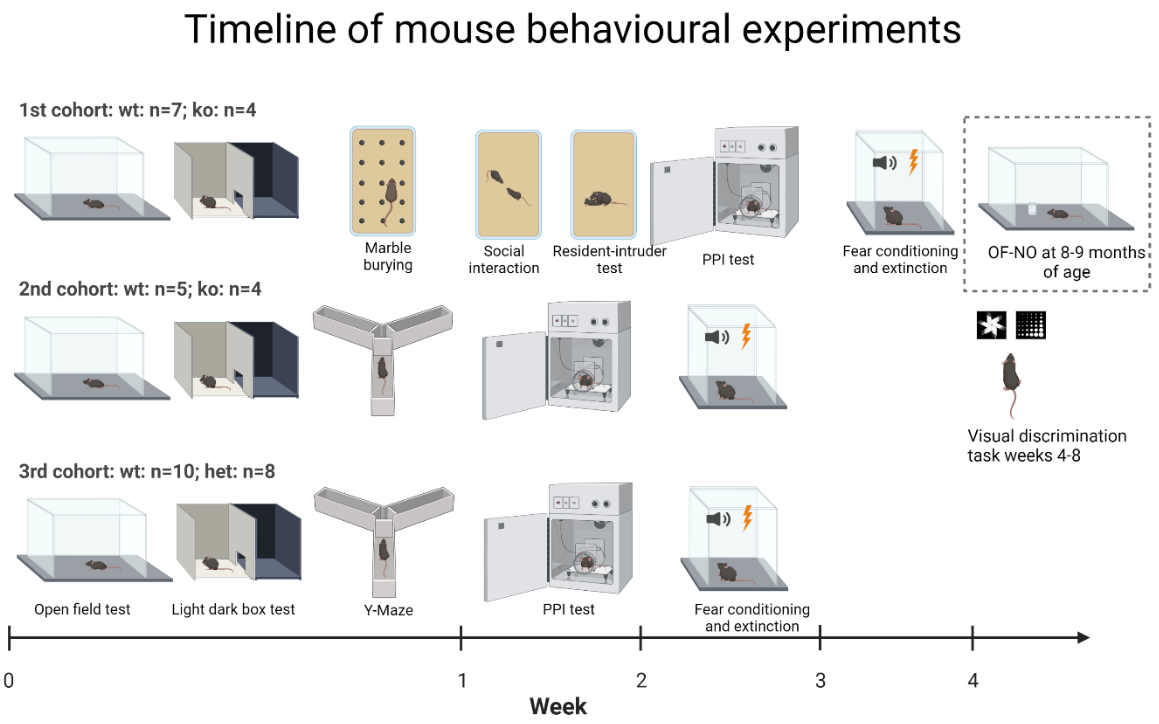

Supplementary Figure 6. Schematic representation of the mouse behavioural experiments. OF/NO – open field/novel object investigation test; PPI – prepulse inhibition test. Created with Biorender.com.
